# Supplementary material for: 1,2,4-Thiadiazolidin-3,5-Diones as Inhibitors of Cysteine Proteases
Source: Molecules. 2025 Sep 26;30(19):3896. doi: 10.3390/molecules30193896 (PMC12526058; doi:10.3390/molecules30193896)
Supplement: Supplementary file 1 [file molecules-30-03896-s001.zip › molecules-3810218-supplementary.docx]

**Supplementary Materials**

1,2,4-Thiadiazolidin-3,5-Diones as Inhibitors of Cysteine Proteases

Maria Aparecida Juliano ^1^, Marco Persico ^2^, Beatrice Severino ^2^, Giuseppe Tumbarello ^2^,
Debora Okamoto ^3^, Karolina Rosa Fernandes ^1^, Gabriel Trigo ^1^, Aparecida Sadae Tanaka ^4^,
José Thalles Lacerda ^1,5^, Oleh Tkachuck ^2^, Angela Corvino ^2^, Ferdinando Fiorino ^2^,
Antonia Scognamiglio ^2^, Francesco Frecentese ^2^, Vincenzo Santagada ^2^, Stefania Vertuccio ^2^,
Giuseppe Caliendo ^2^, Luiz Juliano ^1,^* and Caterina Fattorusso ^2,^*

^1^ Department of Biophysics, Escola Paulista Medicina, Universidade Federal São Paulo, Rua Tres de Maio, 100, São Paulo 04044-020, SP, Brazil; ma.juliano@unifesp.br (M.A.J.); karolina.rosa@unifesp.br (K.R.F.);
gabriel.trigo@unifesp.br (G.T.); thalles_lacerda2@hotmail.com (J.T.L.)

^2^ Department of Pharmacy, School of Medicine, University of Naples «Federico II», Via D. Montesano, 49, 80131 Napoli, Italy; marco.persico@unina.it (M.P.); bseverin@unina.it (B.S.); giuseppe.tumbarello@unina.it (G.T.); oleh.tkachuck@unina.it (O.T.); angela.corvino@unina.it (A.C.);
fefiorin@unina.it (F.F.); antonia.scognamiglio@unina.it (A.S.); francesco.frecentese@unina.it (F.F.); santagad@unina.it (V.S.); stefania.vertuccio@unina.it (S.V.); caliendo@unina.it (G.C.)

^3^ Department of Pharmaceutical Sciences, Universidade Federal de São Paulo, Rua São Nicolau, 210, Diadema 09913-030, SP, Brazil; debora.okamoto@unifesp.br

^4^ Department of Biochemistry, Escola Paulista Medicina, Universidade Federal São Paulo, Rua Tres de Maio, 100, São Paulo 04044-020, SP, Brazil; astanaka10@unifesp.br

^5^  Department of Pharmaceutical Sciences, Federal University of Pernambuco, Rua Artur de Sá, Recife 50740-521, PE, Brazil

***** Correspondence: juliano.luiz@unifesp.br (L.J.); caterina.fattorusso@unina.it (C.F.)

**Table of Contents**

**Tables**

**Table S1:** Initial rates of SARS-CoV2 PLpro using different substrates……………**page S4**

**Table S2.** Values of the kinetic constants for Papain inactivation kinetics analyses by all THIA compounds…………………………………………........................................ **page S5**

**Table S3.** List of amino acids located in the subsites S_1_, S_2_, and S_3_ of 3CLpro, PLpro, Cathepsin L, Papain, and Bromelain. …………….…………………………………………. **page S6**

**Table S4.** ΔE_GM_ values (kcal/mol) and torsional angle values (degrees) of MM and DFT conformers of THIA-1………………………………………………………………….…………… **page S7**

**Table S5.** ΔE_GM_ values (kcal/mol) and torsional angle values (degrees) of MM and DFT conformers of THIA-2. ………………………………….……………………………….………… **page S8**

**Table S6.** ΔE_GM_ values (kcal/mol) and torsional angle values (degrees) of MM and DFT conformers of THIA-3. ………………………………………………………………….………… **page S9**

**Table S7.** ΔE_GM_ values (kcal/mol) and torsional angle values (degrees) of MM and DFT conformers of THIA-4. ………………………………………………………………….………..**page S10**

**Table S8.** ΔE_GM_ values (kcal/mol) and torsional angle values (degrees) of MM and DFT conformers of THIA-5. ………………………………………………………………..………… **page S11**

**Table S9.** ΔE_GM_ values (kcal/mol) and torsional angle values (degrees) of MM and DFT conformers of THIA-6. ……………………………………………………………..…………… **page S12**

**Table S10.** ΔE_GM_ values (kcal/mol) and torsional angle values (degrees) of MM and DFT conformers of THIA-7. ………………………………………………………………..………… **page S13**

**Table S11.** ΔE_GM_ values (kcal/mol) and torsional angle values (degrees) of MM and DFT conformers of THIA-8. ………………………………………………………………..………… **page S14**

**Table S12.** ΔE_GM_ values (kcal/mol) and torsional angle values (degrees) of MM and DFT conformers of THIA-9. …………………………………………………………………..……… **page S15**

**Table S13.** ΔE_GM_ values (kcal/mol) and torsional angle values (degrees) of MM and DFT conformers of THIA-10. ………………………………………………………………………… **page S16**

**Table S14.** Selected 3CLpro and THIA-2 docked complexes………………..…… **page S17**

**Table S15.** DFT conformer, binding mode, ligand-protein non-bond interaction energies (kcal/mol), distance (Å) between the THIA sulfur atom S1 and Cys^145^ sulfur atom of the selected 3CLpro and THIA-4 docked complexes. ………………………………...…… **page S18**

**Table S16.** DFT conformer, binding mode, ligand-protein non-bond interaction energies (kcal/mol), distance (Å) between the THIA sulfur atom S1 and Cys^145^ sulfur atom of the selected 3CLpro and THIA-7 docked complexes. …………………………………...… **page S19**

**Table S17.** DFT conformer, binding mode, ligand-protein non-bond interaction energies (kcal/mol), distance (Å) between the THIA sulfur atom S1 and Cys^145^ sulfur atom of the selected 3CLpro and THIA-8 docked complexes. ………………………………..….… **page S20**

**Table S18.** DFT conformer, binding mode, ligand-protein non-bond interaction energies (kcal/mol), distance (Å) between the THIA sulfur atom S1 and Cys^145^ sulfur atom of the selected 3CLpro and THIA-10 docked complexes. ……………………………..…..… **page S21**

**Table S19.** Summary of Procheck results obtained for the selected docked complexes and the reference structure PDB ID: 7JKV. ……………………………………………… **page S22**

**Table S20.** Ligand-residue nonbonded interaction energies (kcal/mol) of the THIA-2/3CLPro docked complex. …………………………………….………………………….…… **page S23**

**Table S21.** Ligand-residue nonbonded interaction energies (kcal/mol) of the THIA-4/3CLPro docked complex…………………………………………..………….………….…… **page S24**

**Table S22.** Ligand-residue nonbonded interaction energies (kcal/mol) of the THIA-7/3CLPro docked complex…………………………………………..……………………..…… **page S25**

**Table S23.** Ligand-residue nonbonded interaction energies (kcal/mol) of the THIA-8/3CLPro docked complex……………………………………….….……………………..…… **page S26**

**Table S24.** Ligand-residue nonbonded interaction energies (kcal/mol) of the THIA-10/3CLPro docked complex………………………………………..…………………….…..… **page S27**

**Table S25.** Experimentally determined structures of 3CLpro, PLpro, Papain, Cathepsin L, and Bromelain used in the structural and bioinformatics analysis………….……**page S28**

**Table S26.** Solvent accessible surface (SASA) of the sulphur atom of the catalytic cysteine. ………………………………………….……………………………………….…….…… **page S29**

**Figures**

**Figure S1.** (A) - Stability of THIA-3 in DMSO with 1% H2O. (B)- Stability of THIA-3 in water,100 mM sodium phosphate, Tris buffer pH 7.4, with 1 to 40% ACN. … **page S30**

**Figure S2.** Stability of THIAs in dimethyl sulfoxide and stored at -20ºC………. **page S31**

**Figure S3.** Irreversible inhibition of papain, 3CLpro, and reversible inhibition of PLpro by THIA-3………………………………………………………………………………..…………… **page S32**

**Figure S4.** Inhibition of Papain by THIA-3 and reversibility of the activity using cysteine (Cys) as a reducing agent……………………………………………………………..….…… **page S33**

**Figure S5**. Assay with 3CLpro of FRET peptides library having Abz-SAVL**H**SGFRK(Dnp)-NH_2_ as the reference sequence…**…………………………………………………..page S34**

**Figure S6.** Structural comparison between the MM and DFT conformers of THIA-7…………………………………………………………………..….……………………………..…...**page S35**

**Figure S7.** X-ray structure of SARS-CoV-2 3CLpro C145A mutant in complex with substrate Ac-SAVLQSGF-CONH_2_ (PDB ID: 7N89). ……………………………….…… **page S36**

**Figure S8.** X-ray structure of SARS-CoV-2 3CLpro used as protein starting structure in docking studies (PDB ID: 7JKV). …………………………………………………..…..…… **page S37**

**Figure S9.** 3CLPro-THIA best docked complexes superimposed on the starting structure (PDB ID: 7JKV; Cα pairs with a RMSD < 1Å). …………………………………..….…… **page S38**

**Figure S10.** 3CLpro/THIA-6 molecular interaction model obtained by the superimposition of THIA-6 on THIA-4 in the best docked complex with 3CLpro …………………………………………………………………………………………………………….**page S39**

**Figure S11.** Graphs showing the relationship between time (ps) and interaction energy (kcal/mol) calculated considering all the resulting SMD structures……………....**page S40**

**Figure S12**. A) Steered Molecular Dynamics (SMD)results obtained for **THIA-10**. B) Mean Square Fluctuation (RMSF) values calculated considering all the resulting SMD structures………………………………………………………………………………………….…..**page S41**

**Figure S13**. A) Steered Molecular Dynamics (SMD)results obtained for **THIA-2**. B) Mean Square Fluctuation (RMSF) values calculated considering all the resulting SMD structures………………………………………………………………………………………….…..**page S42**

**Figure S14**. A) Steered Molecular Dynamics (SMD)results obtained for **THIA-7**. B) Mean Square Fluctuation (RMSF) values calculated considering all the resulting SMD structures………………………………………………………………………………………….…..**page S43**

**Figure S15**. A) Steered Molecular Dynamics (SMD)results obtained for **THIA-8**. B) Mean Square Fluctuation (RMSF) values calculated considering all the resulting SMD structures………………………………………………………………………………………….…..**page S44**

**Figure S16.** Up: superimposition among x-ray structure of Papain, x-ray structure of Cathepsin L, and x-ray structure of Bromelain. ………………….…….……..….…… **page S45**

**Figure S17.** A: X-ray structure of SARS-CoV-2 PLpro (PDB ID: 6WX4). PLpro is displayed in solid ribbon and coloured in grey………………………………………………..….…… **page S46**

**Figure S18.** X-ray structure of Cathepsin L (PDB ID: 3OF8), Bromelain (PDB ID: 6YCG). Fitting of THIA-2 in the active site of Cathepsin L (PDB ID: 3OF8). Fitting of THIA-2 in the active site of Bromelain (PDB ID: 6YCG)……………………………………….. … **page S47**

**Figure S19.** X-ray structure of SARS-CoV-2 PLpro in complex with a GRL0617 derivative (PDB ID: 7JIW). Fitting of THIA-5 in the allosteric site of PLpro (PDB ID:7JIW). ……………………………………………………………………………………………………..…… **page S48**

**Figure S20.** Superimposition among x-ray structure of Papain (PDB ID: 6TCX), Cathepsin L (PDB ID: 3OF8), and Bromelain (PDB ID: 6YCG). ……………………………………………………………………..……..………..….……………… **page S49**

**Figure S21.** Fitting of THIA-7 in the active site of Bromelain (PDB ID:6YCG). ……………………………………………………………………..………..………..…………..…… **page S50**

**Table S1.** Initial rates of SARS-CoV2 PLpro using different substrates. The arrow (↓) indicates the cleavage site.

Assay conditions: 0.6 μM PLpro incubated with 5 mM DTT for 10 min at 37 °C. 5 μM of each substrate.

**Table S2.** Values of the kinetic constants for Papain inactivation kinetics analyses by all THIA compounds obtained for a two-step irreversible mechanism (see below the reaction paths) as reported in the reference (31)

| **Inhibitors** | **k_3_ (µM.s)^-1^** | **k_4_ (s^-1^)** | **K_i_ (µM)** |
| --- | --- | --- | --- |
| THIA-1 | 19.9 | 0.013 | 0.26 |
| THIA-2 | 24.6 | 0.012 | 0.32 |
| THIA-3 | 19.7 | 0.008 | 0.17 |
| THIA-4 | 4.9 | 0.015 | 0.08 |
| THIA-5 | 12.3 | 0.021 | 0.27 |
| THIA-6 | 14.5 | 0.019 | 0.27 |
| THIA-7 | 17.3 | 0.009 | 0.16 |
| THIA-8 | 109.9 | 0.016 | 0.68 |
| THIA-9 | 14.5 | 0.024 | 0.34 |
| THIA-10 | 13.7 | 0.022 | 0.31 |

Conditions: Papain 48nM, substrate Z-FR-MCA 1.5µM. Buffer: 100 mM sodium phosphate, 1 mM EDTA, pH 6.0. The kinetic constants for the reaction are indicated in the scheme below.


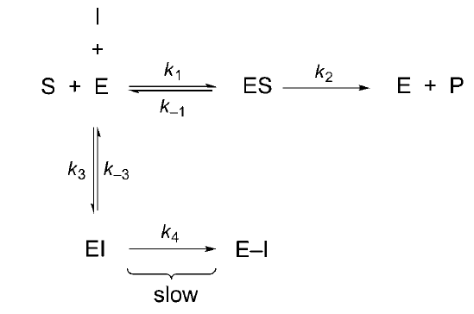


K_i_ represents the relation: K_i_ = k-_3_/k_3_, which is the dissociation constant describing the binding affinity between the inhibitor and the enzyme. In contrast, the IC_50_ is the concentration of inhibitor required to reduce enzymatic activity to half of its uninhibited value. The IC_50_ is less precise than Ki and depends on measurement conditions and the mechanism of inhibition. However, the IC_50_ is convenient for initial characterization of the effects of THIA compounds on cysteine proteases, as it does not require prior mechanistic assumptions, which we addressed through molecular modeling. It is noteworthy that kinetic constants for Papain inactivation by THIA compounds are consistent with a two-step irreversible mechanism.

**Table S3.** List of amino acids located in the subsites S_1_, S_2_ and S_3_ of 3CLpro, PLpro, Cathepsin L, Papain, and Bromelain.

| **Subsite** | **3CLpro** | **PLpro** | **Cathepsin L** | **Papain** | **Bromelain** |
| --- | --- | --- | --- | --- | --- |
| **S_1_** | Phe^140^ | Trp^106^ | Gln^19^ | Gln^19^ | Gln^20^ |
|  | Asn^142^ | Asn^109^ | Gly^23^ | Gly^23^ | Gly^24^ |
|  | Ser^144^ | Cys^111^ | Cys^25^ | Cys^25^ | Cys^26^ |
|  | Cys^145^ | Tyr^112^ | Asp^162^ | Asp^158^ | Asn^157^ |
|  | His^163^ | His^272^ | His^163^ | His^159^ | His^158^ |
|  | His^172^ |  | Trp^189^ | Trp^177^ | Trp^181^ |
| **S_2_** | His^41^ | Leu^162^ | Met^70^ | Pro^68^ | Glu^68^ |
|  | Cys^44^ | Gly^163^ | Ala^135^ | Val^133^ | Ala^133^ |
|  | Met^49^ | Tyr^264^ | Met^161^ | Val^157^ | Leu^156^ |
|  | Pro^52^ | Gly^271^ | Gly^164^ | Ala^160^ | Ala^159^ |
|  | Tyr^54^ | Tyr^273^ | Ala^214^ | Ser^205^ | Asp^209^ |
|  | Met^165^ |  |  |  |  |
|  | Asp^187^ |  |  |  |  |
|  | Gln^189^ |  |  |  |  |
|  | Glu^166^ | Asp^164^ | Gly^61^ | Arg^59^ | Lys^59^ |
|  | Pro^168^ | Tyr^268^ | Asn^62^ | Ser^60^ | Gly^60^ |
| **S_3_** |  | Gln^269^ | Glu^63^ | Tyr^61^ | Tyr^61^ |
|  |  |  | Gly^67^ | Gly^65^ | Gly^65^ |
|  |  |  | Gly^68^ | Gly^66^ | Gly^66^ |
|  |  |  | Leu^69^ | Tyr^67^ | Trp^67^ |
|  |  |  | Tyr^72^ | Ser^70^ | Arg^70^ |

**Table S4.** ΔE_GM_ values (kcal/mol) and torsional angle values (degrees) of MM and DFT conformers of THIA-1.

| **Conf** | **Starting conformer (MM)** | | | | **DFT conformer** | | | |
| --- | --- | --- | --- | --- | --- | --- | --- | --- |
|  | **ΔE_GM_**  **(kcal/mol)** | **τ1^a^** | **τ2^b^** | **τ3^c^** | **ΔE_GM_ (kcal/mol)** | **τ1^a^** | **τ2^b^** | **τ3^c^** |
| 1A | 0.00 | 0° | 88° | 86° | 0.00 | -82° | 82° | 68° |
| 1B | 0.00 | 0° | -88° | -86° | 0.00 | 82° | -82° | -68° |

^a^The values reported refer to the lowest and highest energy conformer of the family. τ1^a^: abcd; τ2^b^: efgh; τ3^c^: fghi.

**Table S5.** ΔE_GM_ values (kcal/mol) and torsional angle values (degrees) of MM and DFT conformers of THIA-2.

| **Conf** | **Starting conformer (MM)** | | | | | **DFT conformer** | | | | |
| --- | --- | --- | --- | --- | --- | --- | --- | --- | --- | --- |
|  | **ΔE_GM_**  **(kcal/mol)** | **τ1^a^** | **τ2^b^** | **τ3^c^** | **τ_OMe_** | **ΔE_GM_ (kcal/mol)** | **τ1^a^** | **τ2^b^** | **τ3^c^** | **τ_OMe_** |
| 1A | 0 | 0° | 88° | 87° | 0° | 0.00 | 90° | 83° | 70° | 0° |
| 1B | 0 | 0° | -88° | -87° | 180° | 0.00 | 90° | -83° | -70° | 180° |
| 2A | 0.05 | 0° | -88° | -87° | 0° | 0.13 | 90° | -82° | -67° | 0° |
| 2B | 0.05 | 0° | 88° | 87° | 180° | 0.13 | 90° | 82° | 67° | 180° |

^a^The values reported refer to the lowest and highest energy conformer of the family. τ1^a^: abcd; τ2^b^: efgh; τ3^c^: fghi; tOMe: jklm.

**Table S6.** ΔE_GM_ values (kcal/mol) and torsional angle values (degrees) of MM and DFT conformers of THIA-3.

| **Conf** | **Starting conformer (MM)** | | | **DFT conformer** | | |
| --- | --- | --- | --- | --- | --- | --- |
|  | **ΔE_GM_ (kcal/mol)** | **τ1^a^** | **τ2^b^** | **ΔE_GM_ (kcal/mol)** | **τ1^a^** | **τ2^b^** |
| 1A | 0 | -9° | 32° | 0.00 | 90° | 90° |
| 1A | 0 | 9° | -32° | 0.00 | 90° | 90° |

^a^The values reported refer to the lowest and highest energy conformer of the family. τ1^a^: abcd; τ2^b^: efgh.

**Table S7.** ΔE_GM_ values (kcal/mol) and torsional angle values (degrees) of MM and DFT conformers of THIA-4.

| **Conf** | **Starting conformer (MM)** | | | | | **DFT conformer** | | | | |
| --- | --- | --- | --- | --- | --- | --- | --- | --- | --- | --- |
|  | **ΔE_GM_ (kcal/mol)** | **τ1^a^** | **τ2^b^** | **τOMe** | **ΔE_GM_ (kcal/mol)** | | **τ1^a^** | **τ2^b^** | **τOMe** |  |
| 1A | 0 | 8° | -32° | 0° | 0.00 | | -88° | 90° | 0° |  |
| 1B | 0 | -8° | 32° | 0° | 0.00 | | 88° | 90° | 180° |  |
| 1A | 0.05 | -9° | 32° | 180° | 0.00 | | -88° | 90° | 0° |  |
| 1B | 0.05 | 9° | -32° | 180° | 0.00 | | 88° | 90° | 180° |  |

^a^The values reported refer to the lowest and highest energy conformer of the family. τ1^a^: abcd; τ2^b^: efgh; τOMe: ijkl

**Table S8.** ΔE_GM_ values (kcal/mol) and torsional angle values (degrees) of MM and DFT conformers of THIA-5.

| **Conf** | **Starting conformer (MM)** | | | | **DFT conformer** | | | |
| --- | --- | --- | --- | --- | --- | --- | --- | --- |
|  | **ΔE_GM_ (kcal/mol)** | **τ1^a^** | **τ2^b^** | **τOMe** | **ΔE_GM_ (kcal/mol)** | **τ1^a^** | **τ2^b^** | **τOMe** |
| 1A | 0 | 9° | -32° | 0° | 0.00 | 90° | 90° | 0° |
| 1B | 0 | -9° | 32° | 0° | 0.00 | 90° | 90° | 180° |
| 1A | 0.05 | -9° | 32° | 180° | 0.00 | 90° | 90° | 0° |
| 1B | 0.05 | -9° | -32° | 180° | 0.00 | 90° | 90° | 180° |

^a^The values reported refer to the lowest and highest energy conformer of the family. τ1^a^: abcd; τ2^b^: efgh; τOMe: ijkl.

**Table S9.** ΔE_GM_ values (kcal/mol) and torsional angle values (degrees) of MM and DFT conformers of THIA-6.

| **Conf** | **Starting conformer (MM)** | | | | | **DFT conformer** | | | | |
| --- | --- | --- | --- | --- | --- | --- | --- | --- | --- | --- |
|  | **ΔE_GM_ (kcal/mol)** | **τ1^a^** | **τ2^b^** | **τOMe** | **τOMe2** | **ΔE_GM_ (kcal/mol)** | **τ1^a^** | **τ2^b^** | **τOMe** | **τOMe2** |
| 1A | 0.00 | 9 | -32 | 0 | 0 | 0.00 | 90° | 90° | 0° | 0° |
| 1B | 0.00 | -9 | 32 | 0 | 0 | 0.00 | 90° | 90° | 180° | 180° |
| 2A | 0.00 | 9 | -32 | 0 | 180° | 0.23 | 90° | 90° | 0° | 180° |
| 2B | 0.00 | -9 | 32 | 0 | 180° | 0.23 | 90° | 90° | 180° | 0° |
| 1A | 0.05 | -9 | 32 | 180° | 180° | 0.00 | 90° | 90° | 0° | 0° |
| 1B | 0.05 | 9 | -32 | 180° | 180° | 0.00 | 90° | 90° | 180° | 180° |
| 2A | 0.05 | 9 | -32 | 180° | 0 | 0.23 | 90° | 90° | 180° | 0° |
| 2B | 0.05 | -9 | 32 | 180° | 0 | 0.23 | 90° | 90° | 0° | 180° |

^a^The values reported refer to the lowest and highest energy conformer of the family. τ1^a^: abcd; τ2^b^: efgh; τOMe: ijkl; τOMe2: mnop

**Table S10.** ΔE_GM_ values (kcal/mol) and torsional angle values (degrees) of MM and DFT conformers of THIA-7.

| **Conf** | **Starting conformer (MM)** | | | | **DFT conformer** | | | |
| --- | --- | --- | --- | --- | --- | --- | --- | --- |
|  | **ΔE_GM_ (kcal/mol)** | **τ1^a^** | **τ2^b^** | **τOMe** | **ΔE_GM_ (kcal/mol)** | **τ1^a^** | **τ2^b^** | **τOMe** |
| 1A | 0 | 8° | -33° | 0° | 0.00 | 90° | 90° | 0 |
| 1B | 0 | -8° | 33° | 0° | 0.00 | 90° | 90° | 180° |
| 1A | 0.05 | -8° | 33° | 180° | 0.00 | 90° | 90° | 0 |
| 1B | 0.05 | 8° | -33° | 180° | 0.00 | 90° | 90° | 180° |

^a^The values reported refer to the lowest and highest energy conformer of the family. τ1^a^: abcd; τ2^b^: efgh; τOMe: ijkl.

**Table S11.** ΔE_GM_ values (kcal/mol) and torsional angle values (degrees) of MM and DFT conformers of THIA-8.

| **Conf** | **Starting conformer (MM)** | | | | **DFT conformer** | | | |
| --- | --- | --- | --- | --- | --- | --- | --- | --- |
|  | **ΔE_GM_ (kcal/mol)** | **τ1^a^** | **τ2^b^** | **τOMe** | **ΔE_GM_ (kcal/mol)** | **τ1^a^** | **τ2^b^** | **τOMe** |
| 1A | 0.33 | 0° | 180° | 0° | 0.00 | 90° | 180° | 0° |
| 1B | 0.38 | 0° | 180° | 180° | 0.00 | 90° | 180° | 180° |
| 2A | 0.05 | 0° | 0° | 0° | 0.72 | 90° | 0° | 0° |
| 2B | 0 | 0° | 0° | 180° | 0.72 | 90° | 0° | 180° |

^a^The values reported refer to the lowest and highest energy conformer of the family. τ1^a^: abcd; τ2^b^: efgh; τOMe: ijkl.

**Table S12.** ΔE_GM_ values (kcal/mol) and torsional angle values (degrees) of MM and DFT conformers of THIA-9.

| **Conf** | **Starting conformer (MM)** | | | **DFT conformer** | | | |
| --- | --- | --- | --- | --- | --- | --- | --- |
|  | **ΔE_GM_ (kcal/mol)** | **τ1^a^** | **τ2^b^** | **ΔE_GM_ (kcal/mol)** | **τ1^a^** | **τ2^b^** |  |
| 1A | 0 | 7° | 148° | 0.00 | 90° | 90° |  |
| 1A | 0 | -7° | -148 | 0.00 | 90° | 90° |  |

^a^ The values reported refer to the lowest and highest energy conformer of the family. τ1^a^: abcd; τ2^b^: efgh.

**Table S13.** ΔE_GM_ values (kcal/mol) and torsional angle values (degrees) of MM and DFT conformers of THIA-10.

| **Conf** | **Starting conformer (MM)** | | | **DFT conformer** | | |
| --- | --- | --- | --- | --- | --- | --- |
|  | **ΔE_GM_ (kcal/mol)** | **τ1^a^** | **τ2^b^** | **ΔE_GM_ (kcal/mol)** | **τ1^a^** | **τ2^b^** |
| 1A | 0 | -10° | +32° | 0.00 | 90° | 90° |
| 1A | 0 | +10° | -32° | 0.00 | 90° | 90° |

^a^The values reported refer to the lowest and highest energy conformer of the family. τ1^a^: abcd; τ2^b^: efgh.

**Table S14.** Selected 3CLpro and THIA-2 docked complexes: ligand conformer, binding mode, ligand-protein non-bond interaction energies (kcal/mol), and distance (Å) between THIA S1 and Cys^145^ sulfur atom.

| Complex | Binding Mode | Nonbonded interaction energies (kcal/mol) | Vdw | Coulomb | Dist S1-S_Cys_ | DFT  conformer |
| --- | --- | --- | --- | --- | --- | --- |
| 1 | II | -38.5438 | -35.2715 | -3.27229 | 3.87211 | 2B |
| 2 | II | -38.2819 | -34.8204 | -3.46146 | 3.76548 | 2B |
| 3 | I | -37.7726 | -37.227 | -0.545614 | 3.43123 | 1B |
| 4 | II | -37.7694 | -35.0276 | -2.74181 | 3.58838 | 1B |
| 5 | I | -37.7428 | -37.2461 | -0.496678 | 3.4514 | 1B |
| 6 | IV | -37.1403 | -38.606 | 1.46566 | 3.34761 | 1A |
| 7 | IV | -37.0753 | -38.5347 | 1.45946 | 3.36116 | 1A |
| 8 | II | -36.0199 | -33.8959 | -2.12396 | 3.77912 | 1B |
| 9 | II | -35.0494 | -31.1844 | -3.865 | 3.55714 | 2A |
| 10 | I | -33.483 | -34.685 | 1.20209 | 3.15277 | 2B |
| 11 | I | -32.504 | -33.538 | 1.03398 | 3.62471 | 2B |
| 12 | I | -32.4709 | -34.1964 | 1.72557 | 3.38585 | 2B |
| 13 | I | -31.9036 | -33.1339 | 1.23033 | 3.93662 | 2A |
| 14 | I | -31.8383 | -33.0723 | 1.23407 | 3.93439 | 2A |
| 15 | III | -30.6344 | -28.6518 | -1.98257 | 3.89249 | 1A |
| 16 | III | -29.2721 | -27.744 | -1.52814 | 3.50433 | 2B |

**Table S15.** DFT conformer, binding mode, ligand-protein non-bond interaction energies (kcal/mol), distance (Å) between the THIA sulfur atom S1 and Cys^145^ sulfur atom of the selected 3CLpro and THIA-4 docked complexes.

| Complex | Binding Mode | Nonbonded interaction energies (kcal/mol) | Vdw | Coulomb | Dist S1-S_Cys_ | DFT  conformer |
| --- | --- | --- | --- | --- | --- | --- |
| 1 | II | -36.5098 | -33.8092 | -2.70059 | 3.59471 | 1B |
| 2 | II | -34.7952 | -32.6152 | -2.17998 | 3.65672 | 1A |
| 3 | I | -30.0629 | -32.5601 | 2.49718 | 3.39406 | 1B |

**Table S16.** DFT conformer, binding mode, ligand-protein non-bond interaction energies (kcal/mol), distance (Å) between the THIA sulfur atom S1 and Cys^145^ sulfur atom of the selected 3CLpro and THIA-7 docked complexes.

| Complex | Binding Mode | Nonbonded interaction energies (kcal/mol) | Vdw | Coulomb | Dist S1-S_Cys_ | DFT conformer |
| --- | --- | --- | --- | --- | --- | --- |
| 1 | II | -37.7838 | -34.8061 | -2.97769 | 3.50877 | 1B |
| 2 | I | -31.0708 | -33.0867 | 2.01594 | 3.33871 | 1B |
| 3 | I | -30.988 | -33.0236 | 2.0356 | 3.3533 | 1B |
| 4 | II | -30.3905 | -28.8106 | -1.57994 | 3.61521 | 1A |
| 5 | III | -29.5137 | -26.6137 | -2.90002 | 3.34087 | 1B |
| 6 | I | -23.5017 | -24.0366 | 0.53488 | 3.91534 | 1A |

**Table S17.** DFT conformer, binding mode, ligand-protein non-bond interaction energies (kcal/mol), distance (Å) between the THIA sulfur atom S1 and Cys^145^ sulfur atom of the selected 3CLpro and THIA-8 docked complexes.

| Complex | Binding Mode | Nonbonded interaction energies (kcal/mol) | Vdw | Coulomb | Dist S1-S_Cys_ | DFT conformer |
| --- | --- | --- | --- | --- | --- | --- |
| 1 | IV | -37.5685 | -38.4296 | 0.861123 | 3.2873 | 2A |
| 2 | II | -36.0927 | -33.9649 | -2.12779 | 3.6883 | 1B |
| 3 | II | -35.8822 | -33.8225 | -2.05961 | 3.72721 | 1B |
| 4 | I | -33.276 | -33.6347 | 0.358675 | 3.18869 | 2B |
| 5 | III | -32.2539 | -30.81 | -1.44392 | 3.27606 | 1B |
| 6 | I | -32.2437 | -33.2754 | 1.03164 | 3.45885 | 1B |
| 7 | I | -31.2258 | -32.4789 | 1.25307 | 3.28696 | 2B |
| 8 | III | -27.8867 | -25.7594 | -2.1273 | 3.82194 | 2A |
| 9 | I | -27.4426 | -28.4937 | 1.05114 | 3.88072 | 2B |
| 10 | III | -26.5165 | -24.409 | -2.1075 | 3.79432 | 2B |

**Table S18.** DFT conformer, binding mode, ligand-protein non-bond interaction energies (kcal/mol), distance (Å) between the THIA sulfur atom S1 and Cys^145^ sulfur atom of the selected 3CLpro and THIA-10 docked complexes.

| Complex | Binding mode | Nonbonded interaction energies (kcal/mol) | Vdw | Coulomb | Dist S1-S_Cys_ | DFT conformer |
| --- | --- | --- | --- | --- | --- | --- |
| 1 | I | -25.5004 | -27.0251 | 1.52468 | 3.05132 | 1A |
| 2 | I | -25.1602 | -26.8279 | 1.66771 | 3.08148 | 1A |

**Table S19.** Summary of Procheck results obtained for the selected docked complexes and the reference structure PDB ID: 7JKV.

| **Complx** | **Binding Mode** | **Residues**  **Favored**  **egions**  **(%)** | **Residues**  **Allowed**  **Regions**  **(%)** | **Residues**  **Generously**  **allowed**  **regions**  **(%)** | **Residues**  **disallowed**  **regions**  **(%)** | **Poor**  **rotamer**  **(%)** |
| --- | --- | --- | --- | --- | --- | --- |
| **THIA-2**/3CLpro | I | 69.8 | 27.9 | 2.1 | 0.2 | 0.6 |
| **THIA-4**/3CLpro | I | 70.9 | 27.0 | 1.9 | 0.2 | 0.3 |
| **THIA-7**/3CLpro | I | 70.7 | 27.2 | 1.9 | 0.2 | 0.3 |
| **THIA-8**/3CLpro | I | 70.7 | 27.0 | 2.1 | 0.2 | 0.3 |
| **THIA-10**/3CLpro | I | 70.6 | 27.2 | 2.1 | 0.2 | 0.3 |
| GRL-2420/3CLpro  (PDB ID: 7JKV) |  | 92.0 | 7.1 | 0.8 | 0.2 | 0.9 |

**Table S20.** Ligand-residue nonbonded interaction energies (kcal/mol) of the THIA-2/3CLPro docked complex (Binding Mode I; residues within 5 Å from any ligand atom).

| ***3CLpro* amino acids** | **Non-bond interaction Energy (kcal/mol)** | | | |
| --- | --- | --- | --- | --- |
|  | **Tot** | **vdW** | **Coulomb** |  |
| His^41^ | -2.427040 | -2.172687 | -0.254353 |  |
| Cys^44^ | -0.101538 | -0.121105 | 0.019567 |  |
| Met^49^ | -2.438598 | -2.644430 | 0.205831 |  |
| Pro^52^ | -0.222867 | -0.192041 | -0.030827 |  |
| Tyr^54^ | -0.350573 | -0.291716 | -0.058857 |  |
| Phe^140^ | -0.407922 | -0.302827 | -0.105095 |  |
| Leu^141^ | -0.204815 | -0.942609 | 0.737794 |  |
| Asn^142^ | -2.964338 | -2.655527 | -0.308811 |  |
| Gly^143^ | -0.249054 | -0.271840 | 0.022786 |  |
| Ser^144^ | -0.260532 | -0.293799 | 0.033267 |  |
| Cys^145^ | -0.839340 | -0.855310 | 0.015970 |  |
| His^163^ | -3.076424 | -1.307997 | -1.768427 |  |
| His^164^ | -1.851120 | -1.930833 | 0.079713 |  |
| Met^165^ | -3.172810 | -3.392754 | 0.219943 |  |
| Glu^166^ | -4.797063 | -6.199283 | 1.402220 |  |
| Leu^167^ | -0.765760 | -0.865254 | 0.099493 |  |
| Pro^168^ | -0.885081 | -0.932440 | 0.047359 |  |
| Val^186^ | -0.181063 | -0.209786 | 0.028724 |  |
| Asp^187^ | -2.791008 | -2.175225 | -0.615783 |  |
| Arg^188^ | -2.860730 | -2.352686 | -0.508044 |  |
| Gln^189^ | -4.660375 | -5.059823 | 0.399448 |  |
| Thr^190^ | -0.392224 | -0.360536 | -0.031688 |  |

**Table S21.** Ligand-residue nonbonded interaction energies (kcal/mol) of the THIA-4/3CLPro docked complex (Binding Mode I; residues within 5 Å from any ligand atom).

| ***3CLpro* amino acids** | **Non-bond interaction Energy (kcal/mol)** | | |
| --- | --- | --- | --- |
|  | **Tot** | **vdW** | **Coulomb** |
| His^41^ | -1.891076 | -1.929486 | 0.038409 |
| Cys^44^ | -0.129054 | -0.140748 | 0.011693 |
| Met^49^ | -2.526046 | -2.743346 | 0.217300 |
| Leu^50^ | -0.201198 | -0.173523 | -0.027675 |
| Pro^52^ | -0.224814 | -0.217374 | -0.007440 |
| Tyr^54^ | -0.617443 | -0.475845 | -0.141598 |
| Leu^141^ | -0.018327 | -0.591218 | 0.572890 |
| Asn^142^ | -1.626461 | -2.668457 | 1.041996 |
| Gly^143^ | -0.169751 | -0.176251 | 0.006500 |
| Cys^145^ | -0.517387 | -0.522814 | 0.005427 |
| His^163^ | -1.106671 | -0.556519 | -0.550152 |
| His^164^ | -1.698157 | -1.786646 | 0.088490 |
| Met^165^ | -2.672809 | -3.081361 | 0.408552 |
| Glu^166^ | -3.435596 | -5.297094 | 1.861499 |
| Leu^167^ | -0.631097 | -0.587567 | -0.043530 |
| Pro^168^ | -0.257379 | -0.297057 | 0.039679 |
| Val^186^ | -0.203572 | -0.190641 | -0.012931 |
| Asp^187^ | -3.205367 | -2.705842 | -0.499524 |
| Arg^188^ | -2.690873 | -2.720347 | 0.029474 |
| Gln^189^ | -3.698229 | -3.572302 | -0.125927 |
| Thr^190^ | -0.429669 | -0.398122 | -0.031547 |

**Table S22.** Ligand-residue nonbonded interaction energies (kcal/mol) of the THIA-7/3CLPro docked complex (Binding Mode I; residues within 5 Å from any ligand atom).

| ***3CLpro* amino acids** | **Non-bond interaction Energy (kcal/mol)** | | |
| --- | --- | --- | --- |
|  | **Tot** | **vdW** | **Coulomb** |
| His^41^ | -1.827870 | -1.801248 | -0.026622 |
| Cys^44^ | -0.120338 | -0.137494 | 0.017155 |
| Met^49^ | -2.464100 | -2.672417 | 0.208317 |
| Leu^50^ | -0.202179 | -0.169243 | -0.032936 |
| Pro^52^ | -0.222285 | -0.208204 | -0.014081 |
| Tyr^54^ | -0.574012 | -0.443705 | -0.130307 |
| Leu^141^ | -0.120080 | -0.612712 | 0.492632 |
| Asn^142^ | -2.134234 | -2.773782 | 0.639548 |
| Gly^143^ | -0.203762 | -0.205127 | 0.001364 |
| Ser^144^ | -0.215778 | -0.196214 | -0.019564 |
| Cys^145^ | -0.527392 | -0.546379 | 0.018987 |
| His^163^ | -1.044910 | -0.694164 | -0.350745 |
| His^164^ | -1.685335 | -1.795693 | 0.110358 |
| Met^165^ | -2.774140 | -3.234895 | 0.460755 |
| Glu^166^ | -3.596628 | -5.234693 | 1.638065 |
| Leu^167^ | -0.527712 | -0.540813 | 0.013100 |
| Pro^168^ | -0.630213 | -0.654719 | 0.024506 |
| Val^186^ | -0.249250 | -0.216945 | -0.032305 |
| Asp^187^ | -3.171744 | -2.670339 | -0.501405 |
| Arg^188^ | -2.684517 | -2.662936 | -0.021581 |
| Gln^189^ | -3.675208 | -3.603570 | -0.071638 |
| Thr^190^ | -0.483076 | -0.417743 | -0.065334 |

**Table S23.** Ligand-residue nonbonded interaction energies (kcal/mol) of the THIA-8/3CLPro docked complex (Binding Mode I; residues within 5 Å from any ligand atom).

| ***3CLpro* amino acids** | **Non-bond interaction Energy (kcal/mol)** | | |
| --- | --- | --- | --- |
|  | **Tot** | **vdW** | **Coulomb** |
| His^41^ | -2.306385 | -2.342379 | 0.035994 |
| Cys^44^ | -0.136183 | -0.141520 | 0.005337 |
| Met^49^ | -2.661530 | -2.793487 | 0.131958 |
| Leu^50^ | -0.190469 | -0.177977 | -0.012491 |
| Pro^52^ | -0.229519 | -0.236559 | 0.007040 |
| Tyr^54^ | -0.674134 | -0.514778 | -0.159357 |
| Leu^141^ | 0.030413 | -0.431842 | 0.462255 |
| Asn^142^ | -2.849585 | -2.810907 | -0.038678 |
| Gly^143^ | -0.340767 | -0.274931 | -0.065836 |
| Ser^144^ | -0.404543 | -0.251801 | -0.152742 |
| Cys^145^ | -0.384145 | -0.438149 | 0.054003 |
| His^163^ | -1.110454 | -0.578537 | -0.531917 |
| His^164^ | -1.741687 | -1.780925 | 0.039238 |
| Met^165^ | -2.600214 | -2.985904 | 0.385691 |
| Glu^166^ | -4.600050 | -5.484631 | 0.884581 |
| Leu^167^ | -0.646818 | -0.616097 | -0.030721 |
| Pro^168^ | -0.327598 | -0.353060 | 0.025462 |
| Val^186^ | -0.196280 | -0.184947 | -0.011333 |
| Asp^187^ | -3.187854 | -2.677634 | -0.510220 |
| Arg^188^ | -2.599143 | -2.685730 | 0.086588 |
| Gln^189^ | -3.813559 | -3.857426 | 0.043868 |
| Thr^190^ | -0.434878 | -0.418323 | -0.016555 |

**Table S24.** Ligand-residue nonbonded interaction energies (kcal/mol) of the THIA-10/3CLPro docked complex (Binding Mode I; residues within 5 Å from any ligand atom).

| ***3CLpro* amino acids** | **Non-bond interaction Energy (kcal/mol)** | | | |
| --- | --- | --- | --- | --- |
|  | **Tot** | **vdW** | **Coulomb** |  |
| His^41^ | -2.983289 | -2.901081 | -0.082208 |  |
| Met^49^ | -1.659748 | -1.921241 | 0.261493 |  |
| Tyr^54^ | -0.193688 | -0.191025 | -0.002664 |  |
| Leu^141^ | -0.168642 | -0.371204 | 0.202562 |  |
| Asn^142^ | -2.352808 | -3.104485 | 0.751676 |  |
| Gly^143^ | -0.366503 | -0.258800 | -0.107703 |  |
| Ser^144^ | -0.294487 | -0.169366 | -0.125121 |  |
| Cys^145^ | -0.191843 | -0.215337 | 0.023494 |  |
| His^163^ | -0.654949 | -0.441345 | -0.213604 |  |
| His^164^ | -1.797310 | -1.799533 | 0.002223 |  |
| Met^165^ | -2.577079 | -2.962116 | 0.385037 |  |
| Glu^166^ | -2.586172 | -3.427047 | 0.840875 |  |
| Leu^167^ | -0.494232 | -0.465337 | -0.028894 |  |
| Pro^168^ | -0.268050 | -0.224777 | -0.043273 |  |
| Val^186^ | -0.160107 | -0.170034 | 0.009927 |  |
| Asp^187^ | -1.041910 | -1.114112 | 0.072202 |  |
| Arg^188^ | -1.096935 | -1.224645 | 0.127710 |  |
| Gln^189^ | -4.413785 | -3.875031 | -0.538753 |  |
| Thr^190^ | -0.467475 | -0.438970 | -0.028505 |  |

**Table S25.** Experimentally determined structures of 3CLpro, PLpro, Papain, Cathepsin L, and Bromelain we employed in the structural and bioinformatics analysis.

| **PDB ID** | **Organism** | **Structure** | **Resolution (Å)** |
| --- | --- | --- | --- |
| 7JKV | SarsCoV2 | Crystal Structure of SARS-CoV-2 main protease in complex with the inhibitor GRL-2420 | 1.25 |
| 7N89 | SarsCoV2 | X-ray structure of SARS-CoV-2 main protease C145A mutant in complex with substrate Ac-SAVLQSGF-CONH2 | 2.00 |
| 6WX4 | SarsCoV2 | Crystal structure of the SARS CoV-2 Papain-like protease in complex with peptide inhibitor VIR251 | 1.66 |
| 7JIW | SarsCoV2 | The crystal structure of Papain-Like Protease of SARS CoV-2 in complex with PLP_Snyder530 inhibitor | 2.30 |
| 3OF8 | H.Sapiens | Structural Basis for Reversible and Irreversible Inhibition of Human Cathepsin L by their Respective Dipeptidyl Glyoxal and Diazomethylketone Inhibitors | 2.20 |
| 6TCX | Papaya | Papain bound to a natural cysteine protease inhibitor from Streptomyces mobaraensis | 1.65 |
| 6YCG | Ananas comosus | Structure of the bromelain protease from Ananas comosus in complex with the TLCK inhibitor | 1.45 |

**Table S26.** Solvent accessible surface (SASA) of the sulphur atom of the catalytic cysteine.

| **PDB ID** | **Structure** | **Solvent accessible surface (SASA)**  **(Å^2^)** |
| --- | --- | --- |
| 7JKV | 3CLpro | 9.668 |
| 6WX4 | PLpro | 6.732 |
| 6TCX | Papain | 14.055 |
| 3OF8 | Cathepsin L | 14.135 |
| 6YCG | Bromelain | 15.548 |

**FIGURES**

**(A)**

| **Time = 0** | **Time = 1h** | **Time = 24 h** | **Time = 48 h** |
| --- | --- | --- | --- |
|  |  |  |  |
| 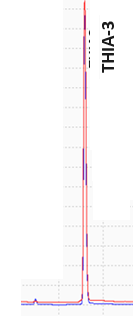 | 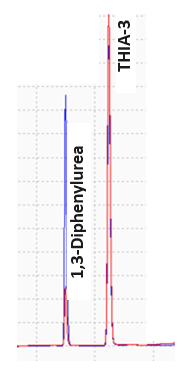 | 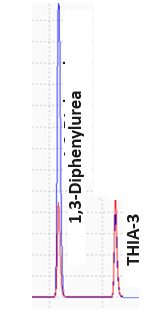 | 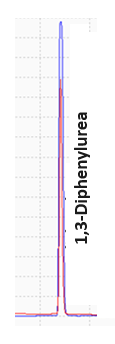 |

**(B)**

| **ACN(40%)/H_2_O (60%) after 48 h** | **Phosphate Buffer, pH 7.4 with 5% ACN.** | **Tris Buffer, pH 7.4 with 1% ACN** |
| --- | --- | --- |
| 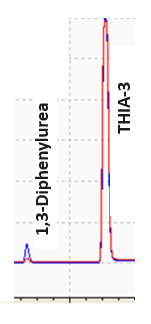 | 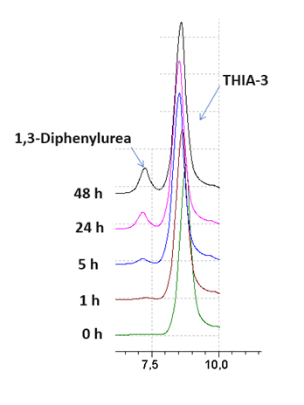 | 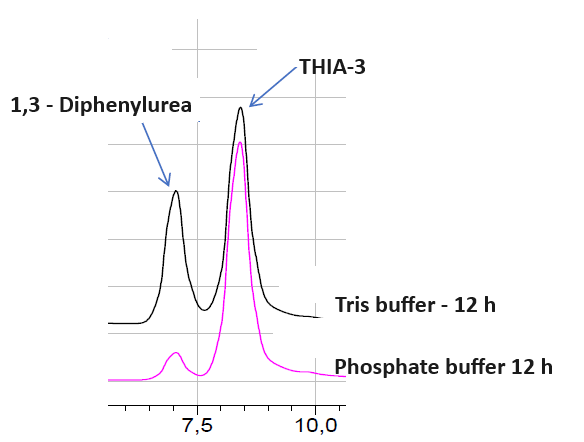 |

**Figure S1.** (A) - Stability THIA-3 in DMSO with 1% H2O. (B)- Stability of THIA-3 in water,100 mM sodium phosphate, Tris buffer pH 7.4, with 1 to 40% ACN.

| Chromatographic profile | MS Spectrum Degradation Product | Chromatographic profile | MS Spectrum Degradation Product |
| --- | --- | --- | --- |
| THIA-1  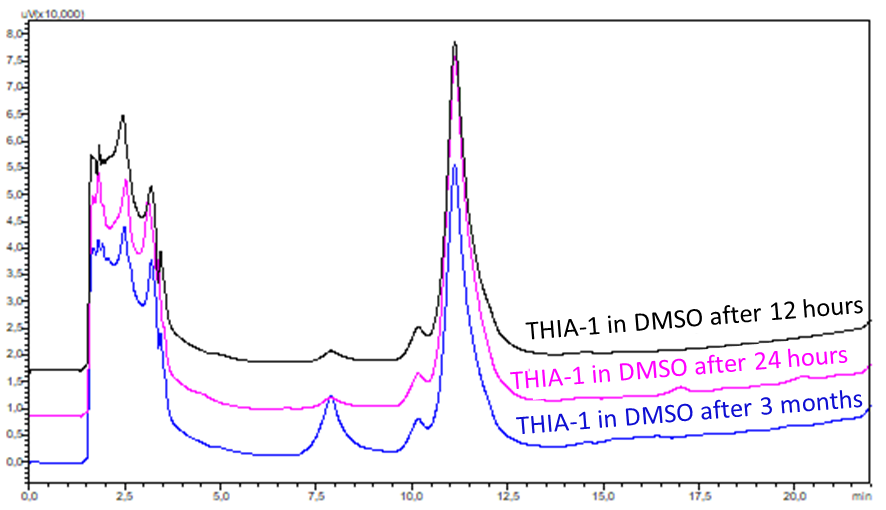 | THIA-1  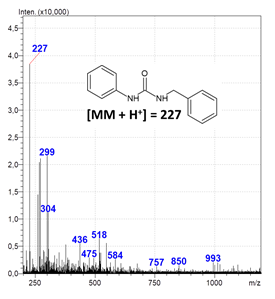 | THIA-6  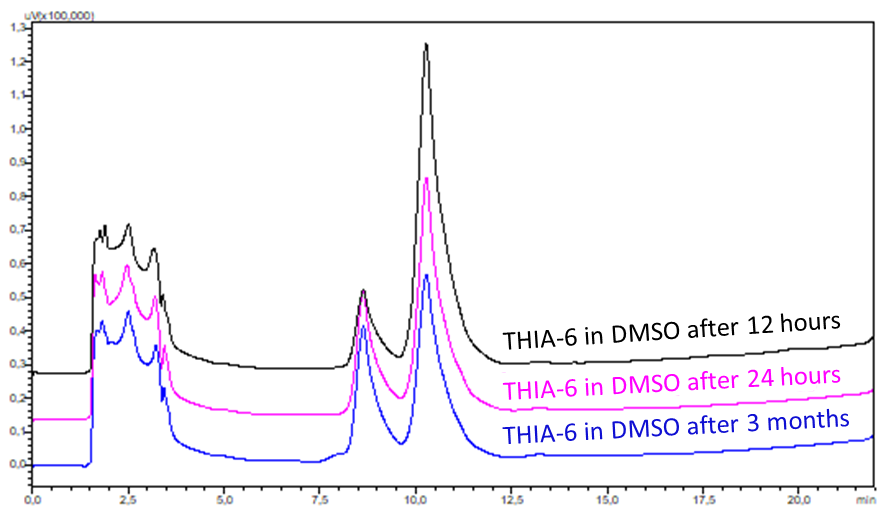 | THIA-6  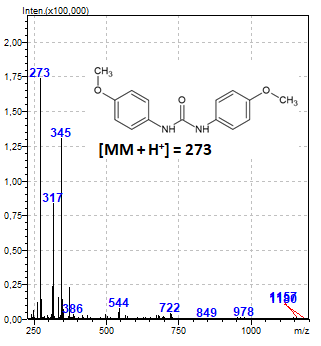 |
| THIA-2  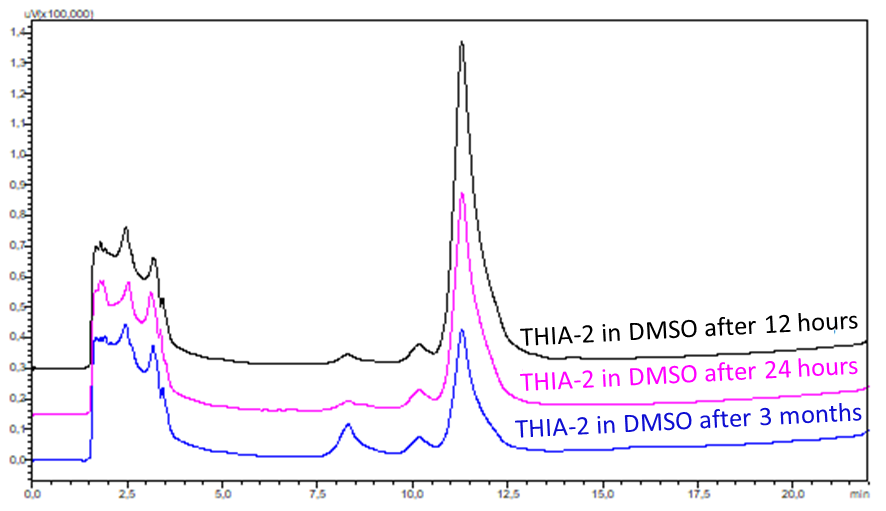 | THIA-2  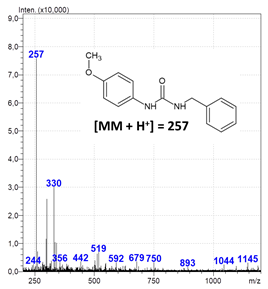 | THIA-7  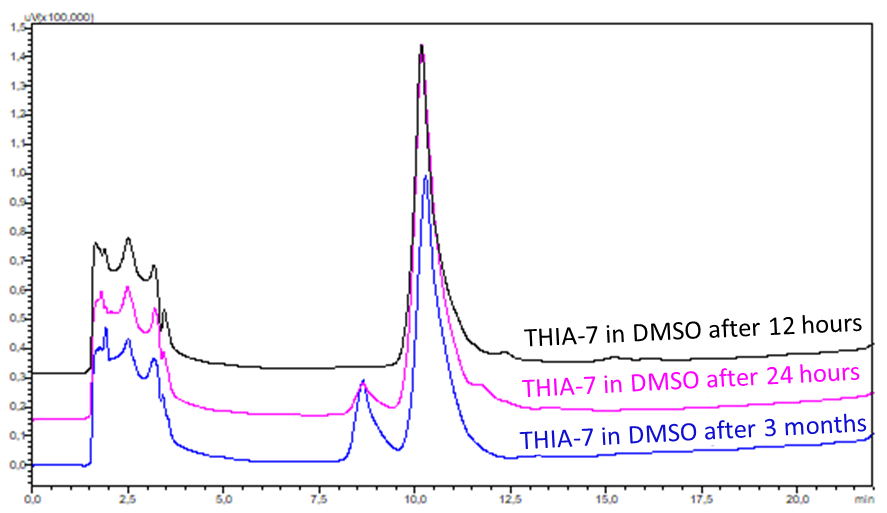 | THIA-7  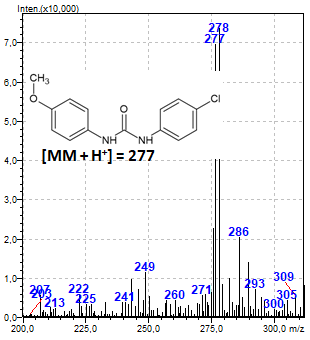 |
| THIA-3  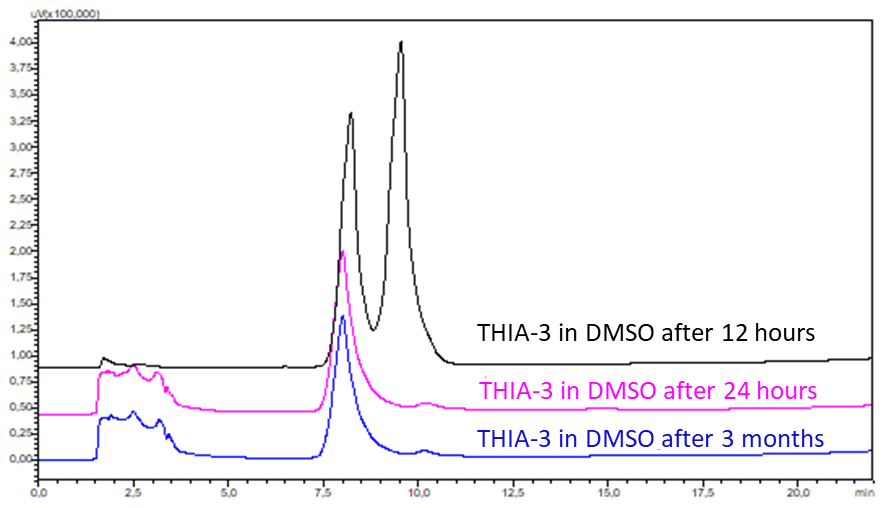 | THIA-3  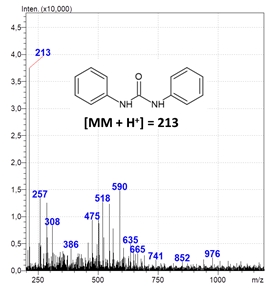 | THIA-8  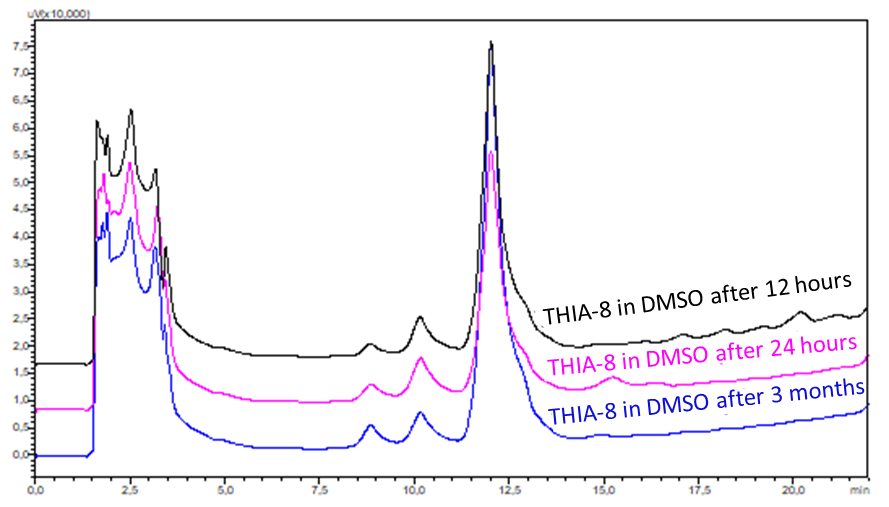 | THIA-8  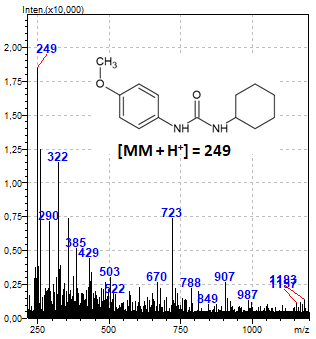 |
| THIA-4  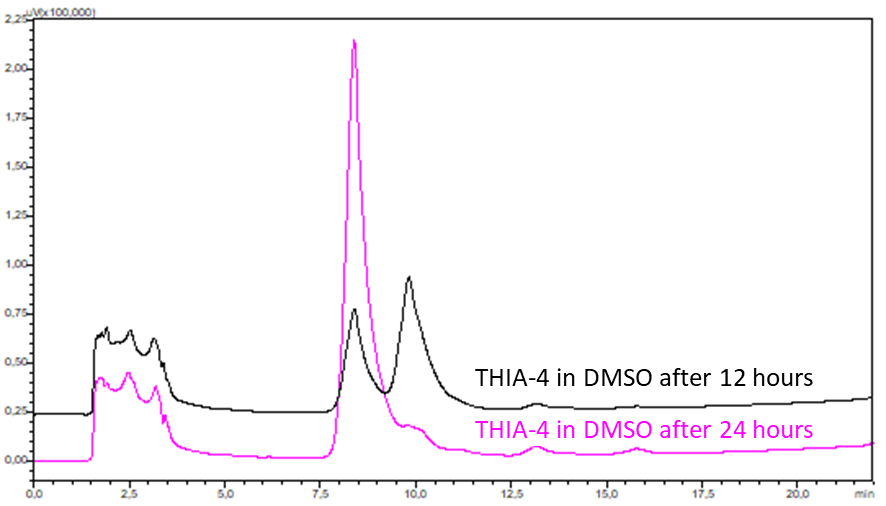 | THIA-4  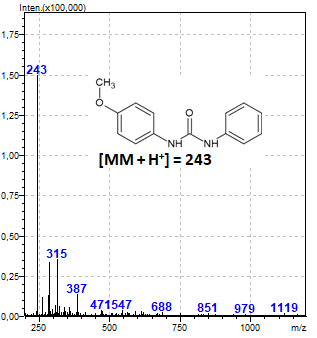 | THIA-9  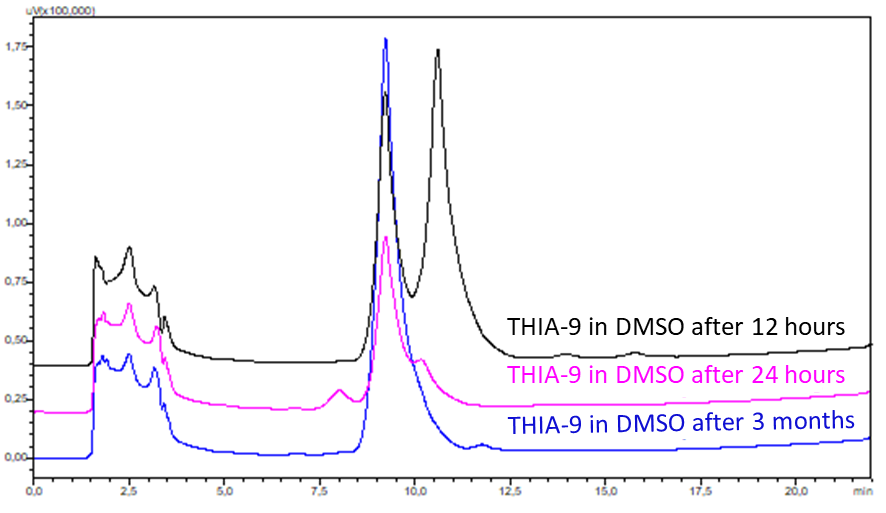 | THIA-9  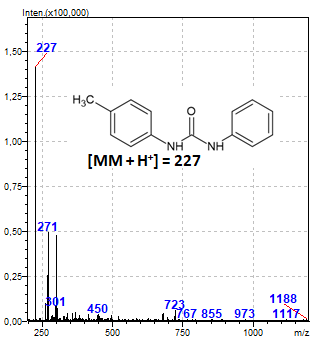 |
| THIA-5  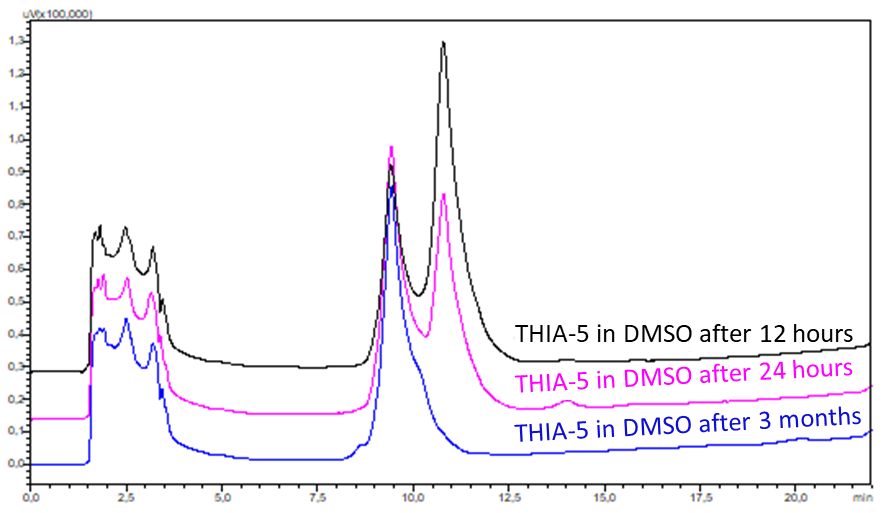 | THIA-5  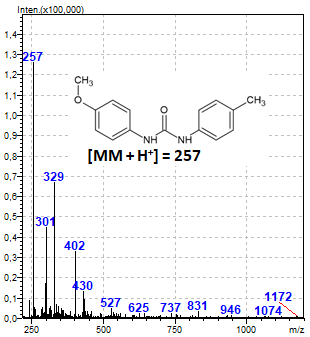 | THIA-10  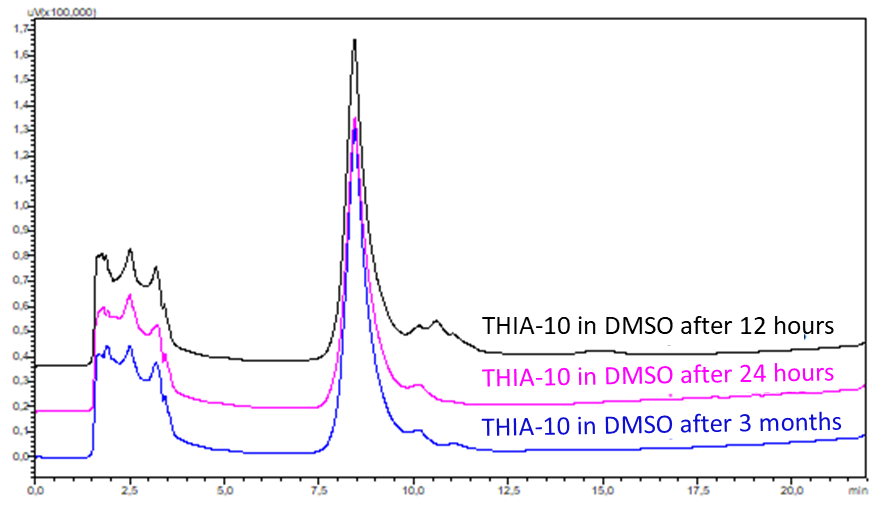 | THIA-10  **No degradation detected** |

**Figure S2.** Stability of THIAs in dimethyl sulfoxide and stored at -20º C

A) Papain


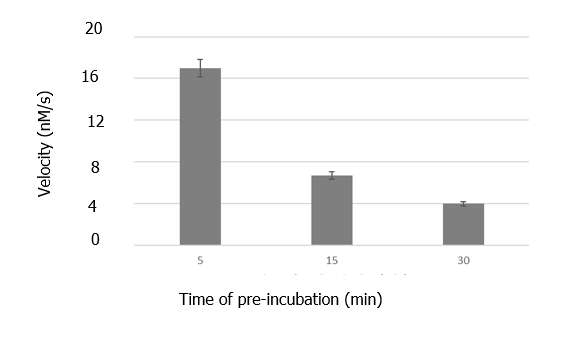


B) 3CLpro


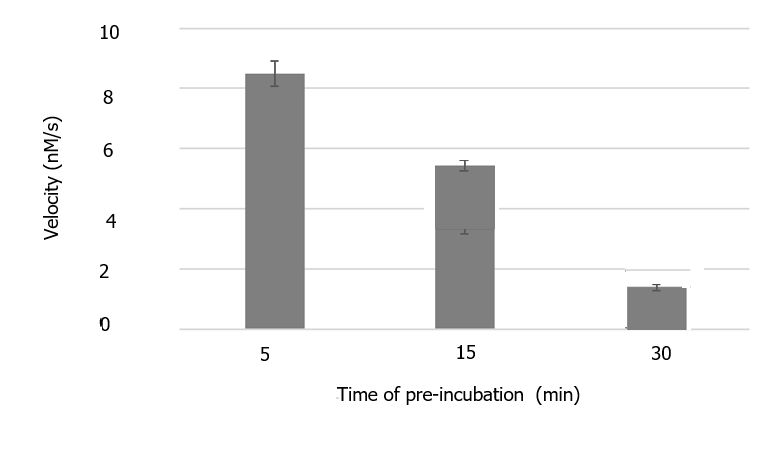


Assay for reversibility/irreversibility


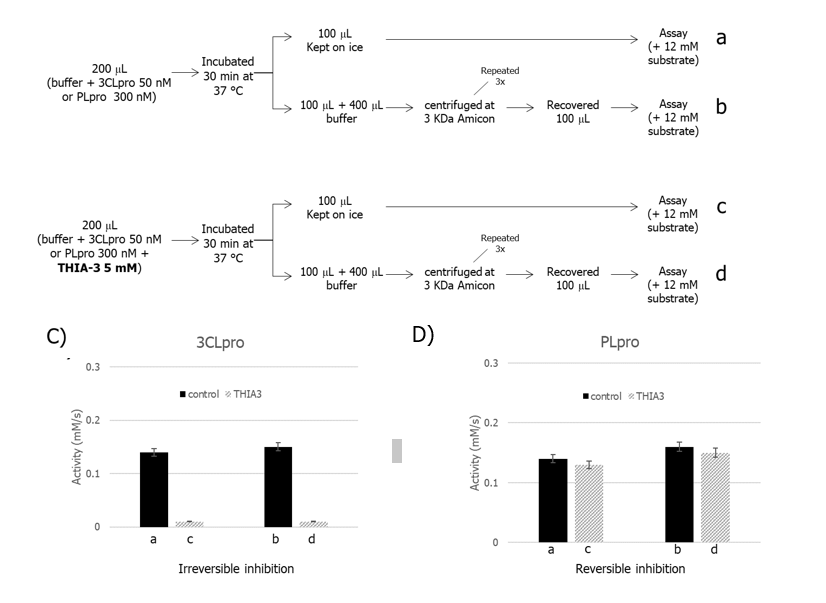


**Figure S3.** Time-dependent inhibition of papain (A) and 3CLpro (B). Assay of reversibility/ irreversibility of inhibition for 3CLpro (C) and PLpro (D), and reversible inhibition of PLpro (C) by THIA-3. The assay has been performed following the procedure described in Reference [30] ACS Omega 6 (2021) 16584−16591. <https://doi.org/10.1021/acsomega.1c01797>.


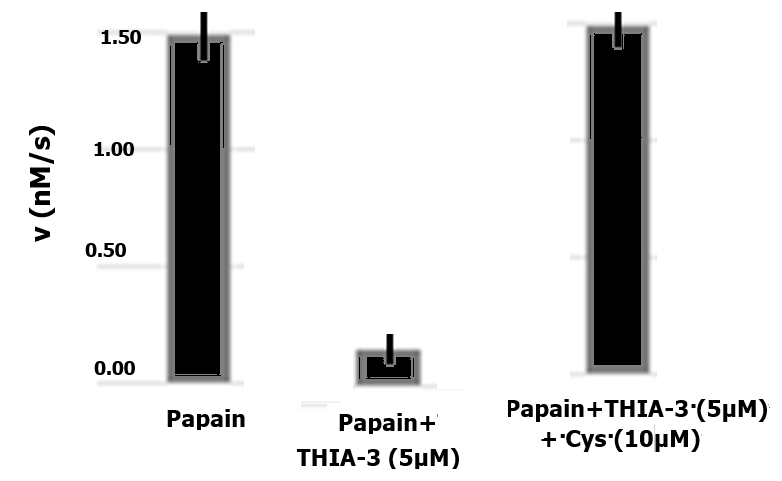


**Figure S4.** Inhibition of Papain by THIA-3 and its reversibility by cysteine (Cys) as a reducing agent. Z-FR-MCA as substrate, Papain activity was assessed before and after pre-incubation with 5µM THIA-3 for 5 min, and then assayed after Cys addition (10 µM). These data are the average of three experiments, and bars are the standard errors.

**
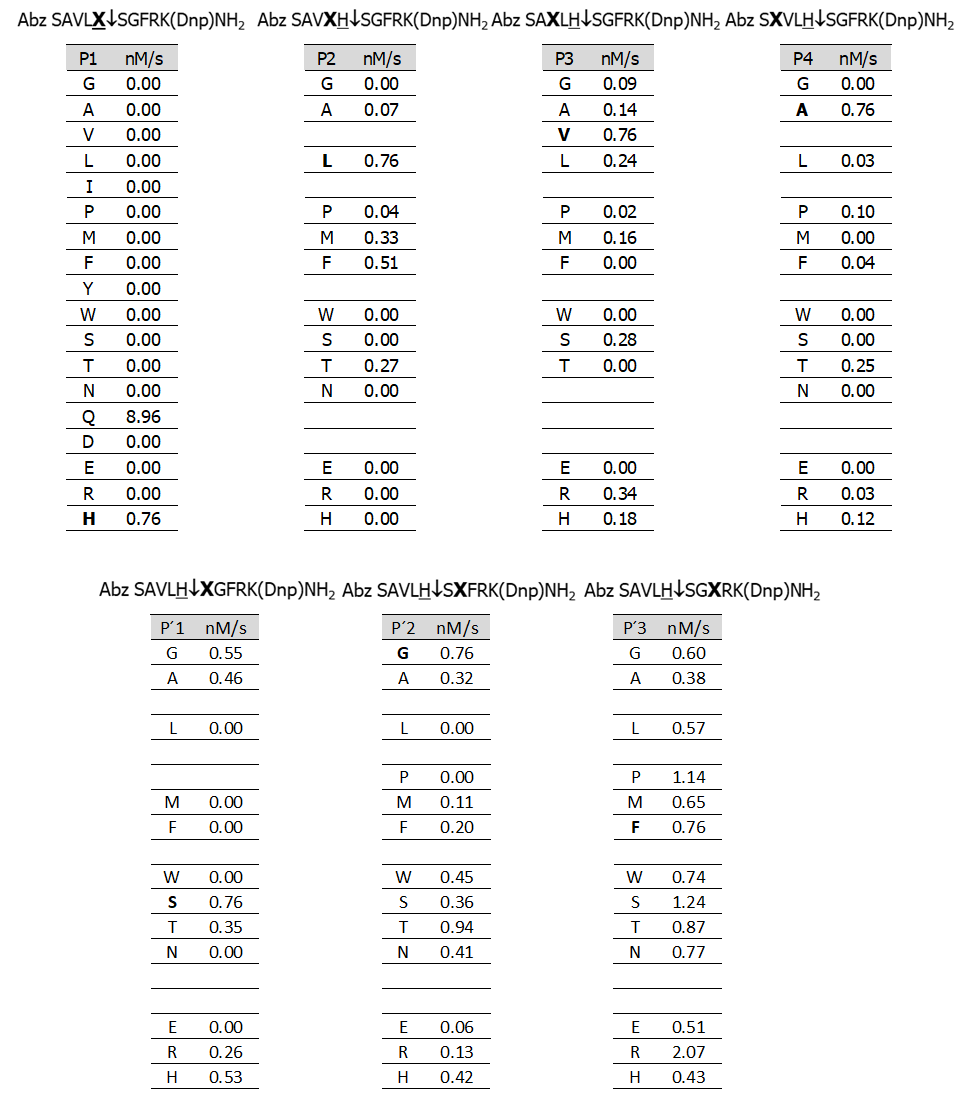
**

**Figure S5**. Assay with 3CLpro of FRET peptides library having Abz-SAVL**H**SGFRK(Dnp)-NH_2_ as the reference sequence. Conditions: 50mM Tris, 1mM EDTA pH 7.5, 5mM DTT for at 37C. Concentrations: [3CLpro] = 54 nM, [Peptides]= 12 µM


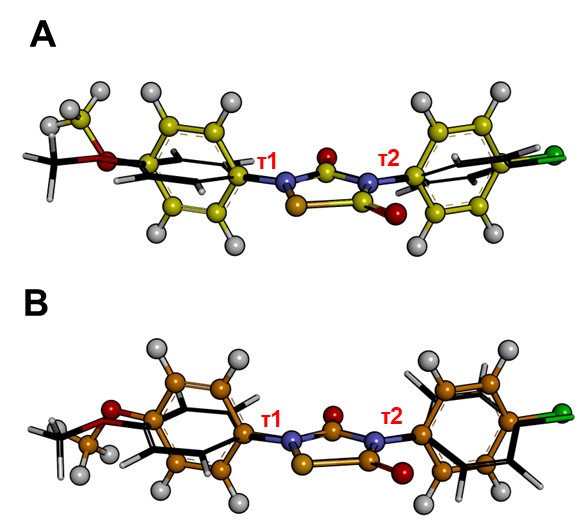


**Figure S6.** Structural comparison between the MM (stick; black) and DFT (ball& stick) conformers of THIA-7. The conformational enantiomer A is shown in (A) while the conformational enantiomer B in (B). The ligands are coloured by atom type: O=red; N=blue; S=yellow; Cl=green; H=white.

**
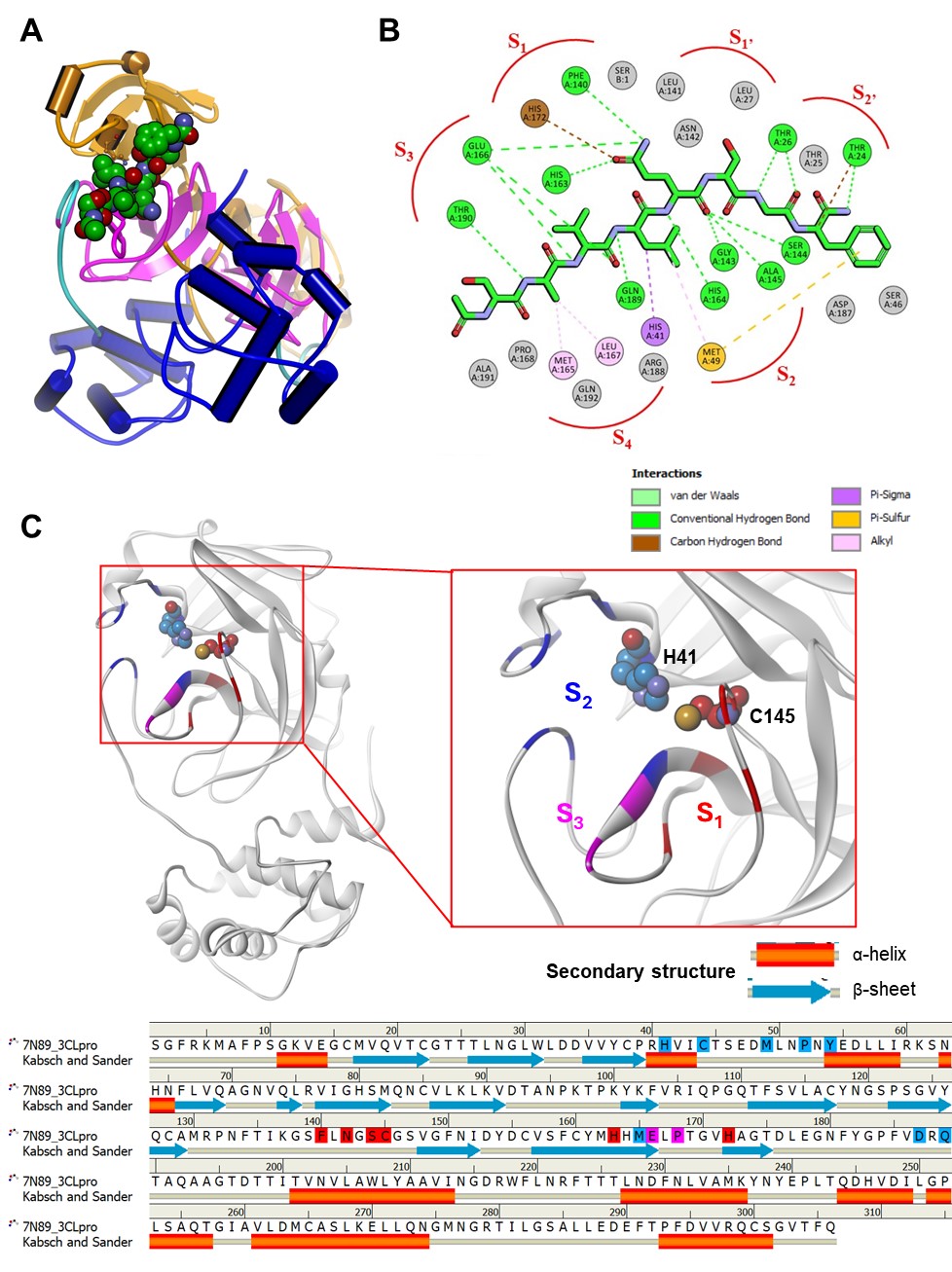
**

**Figure S7.** A: X-ray structure of SARS-CoV-2 3CLpro C145A mutant in complex with substrate Ac-SAVLQSGF-CONH_2_ (PDB ID: 7N89). 3CLpro is coloured by protein domains: Domain I is coloured in orange; Domain II in magenta, LH-loop in cyan, and Domain III in blue. The secondary structure is displayed as wide cylinders (α-helices), arrows (beta-sheets), and tubes (coils and turns). The substrate is displayed in CPK and coloured by atom type (C= green, O = red, N = blue). B: schematic representation of the interactions between substrate and 3CLpro. All residues within 5 Å of any ligand atom are displayed. C: 3CLpro is displayed in solid ribbon and colored in grey. The ribbon of residues of S_1_, S_2,_ and S_3_ subsites is coloured in red, blue, and magenta, respectively. The catalytic residues Cys and His are displayed in CPK and labelled. The sequence and the secondary structure assignments (Kabsch and Sander) are reported.


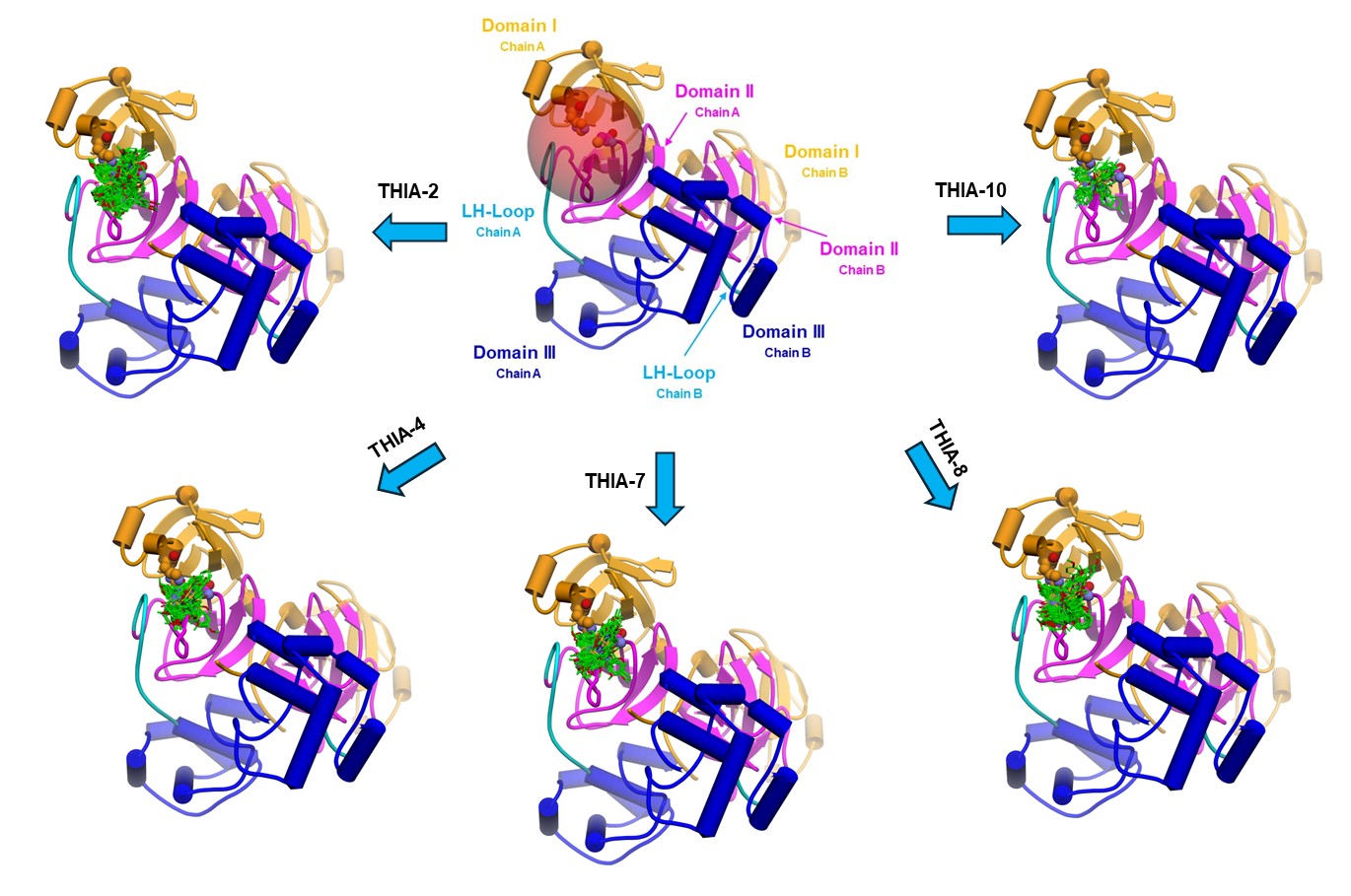


**Figure S8.** X-ray structure of SARS-CoV-2 3CLpro used as protein starting structure in docking studies (PDB ID: 7JKV). The search area defined for ligand docking is represented as a transparent red sphere. The secondary structure is displayed as wide cylinders (α-helices), arrows (beta-sheets), and tubes (coils and turns), and is coloured by protein domains: Domain I of each monomer is coloured in orange; Domain II in magenta, LH-loop in cyan, and Domain III in blue. THIA-2, THIA-4, THIA-7, THIA-8, and THIA-10 binding poses resulting from docking studies are shown. The ligands are displayed in stick and coloured by atom type (N=blue; O=red; S=yellow; Cl=green; F= cyan).

**
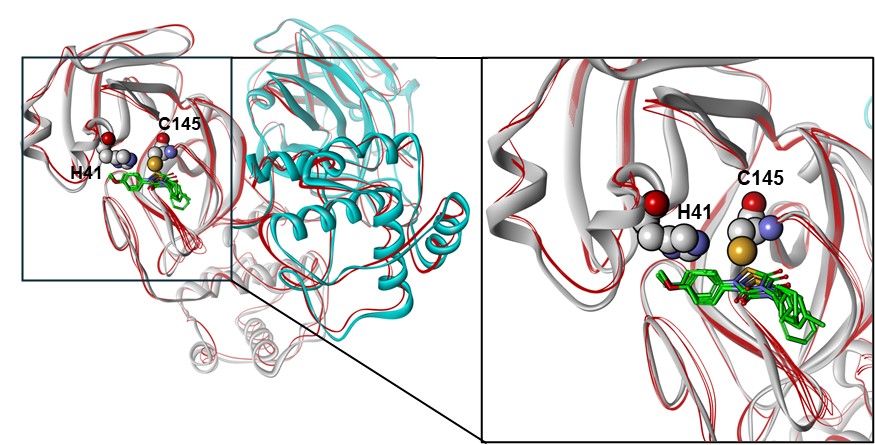
**

**Figure S9.** 3CLPro-THIA best docked complexes superimposed on the starting structure (PDB ID: 7JKV; Cα pairs with a RMSD < 1Å). The backbone of the starting complex is displayed as solid ribbon and coloured in grey monomer A and cyan monomer B. The backbone of the calculated complexes is displayed as line ribbons and coloured in red. The ligands ( are coloured by atom type (C: green; N: blue; S: yellow; O: red) and displayed in stick. The two catalytic residues are shown in CPK and labelled.


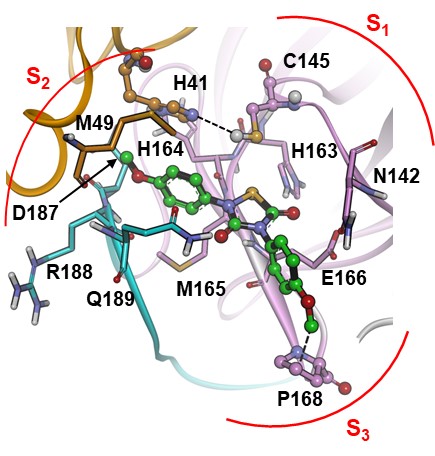


**Figure S10.** 3CLpro/THIA-6 molecular interaction model obtained by the superimposition of THIA-6 (ball&stick; green) on THIA-4 (stick; black) in the best docked complex with 3CLpro. The protein is displayed as solid ribbons and coloured by protein domains: orange (Domain I: aa 1-101), pink (Domain II: aa 102-184), and cyan (LH-Loop: aa 185-200). The residues involved in the interactions with the ligand are displayed in sticks; the catalytic residues His^41^ and Cys^145^ as well as Pro^168^ are displayed in ball&stick and the putative hydrophobic interaction of THIA-6 with Pro^168^ is evidenced by black dashed lines.


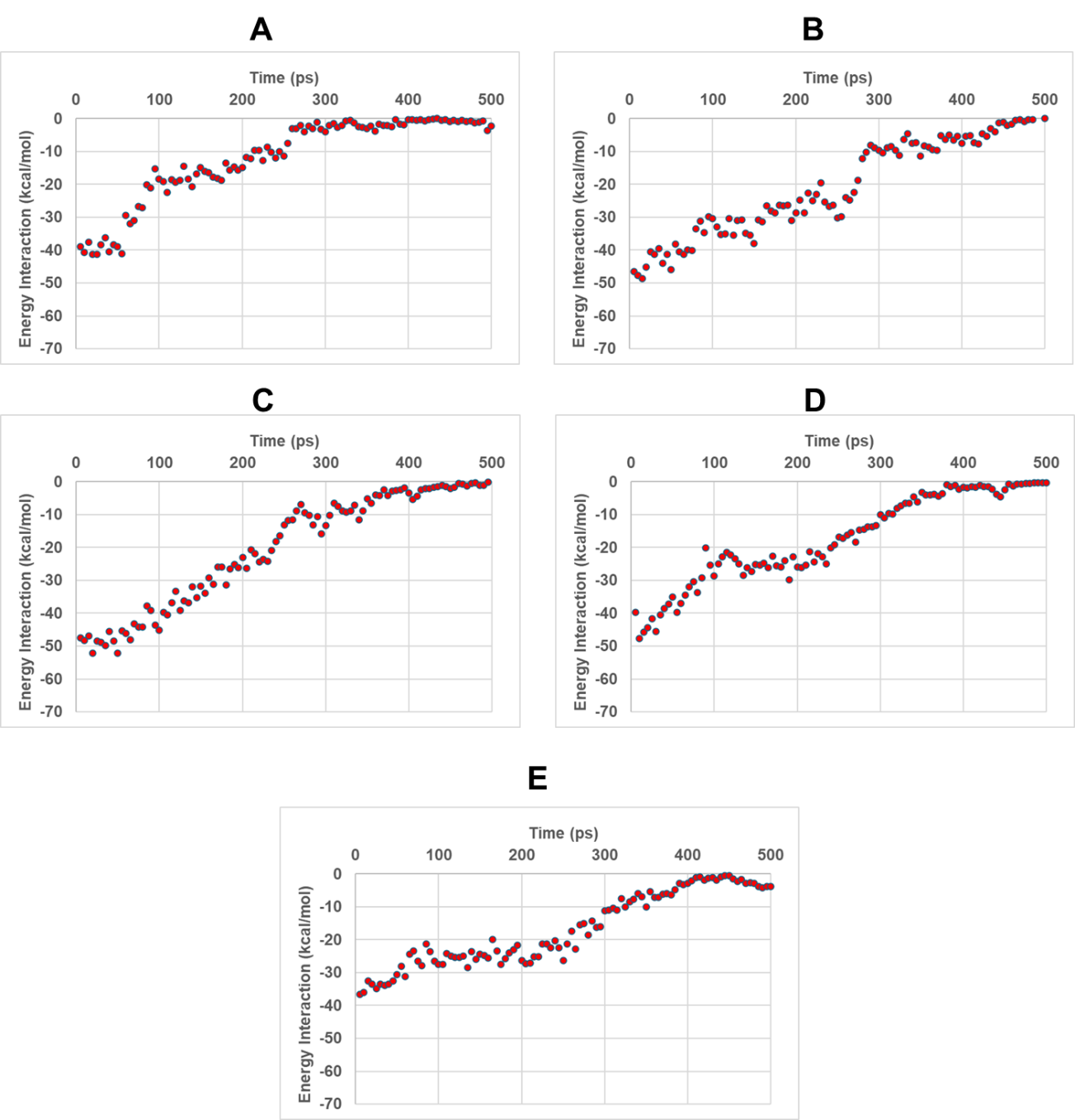


**Figure S11.** Graphs showing the relationship between time (ps) and interaction energy (kcal/mol) calculated considering all the resulting SMD structures of THIA-10 (A), THIA-2 (B), THIA-4 (C), THIA-7 (D) and THIA-8 (E). Red dots represent interaction energy values calculated every 5 ps.

**
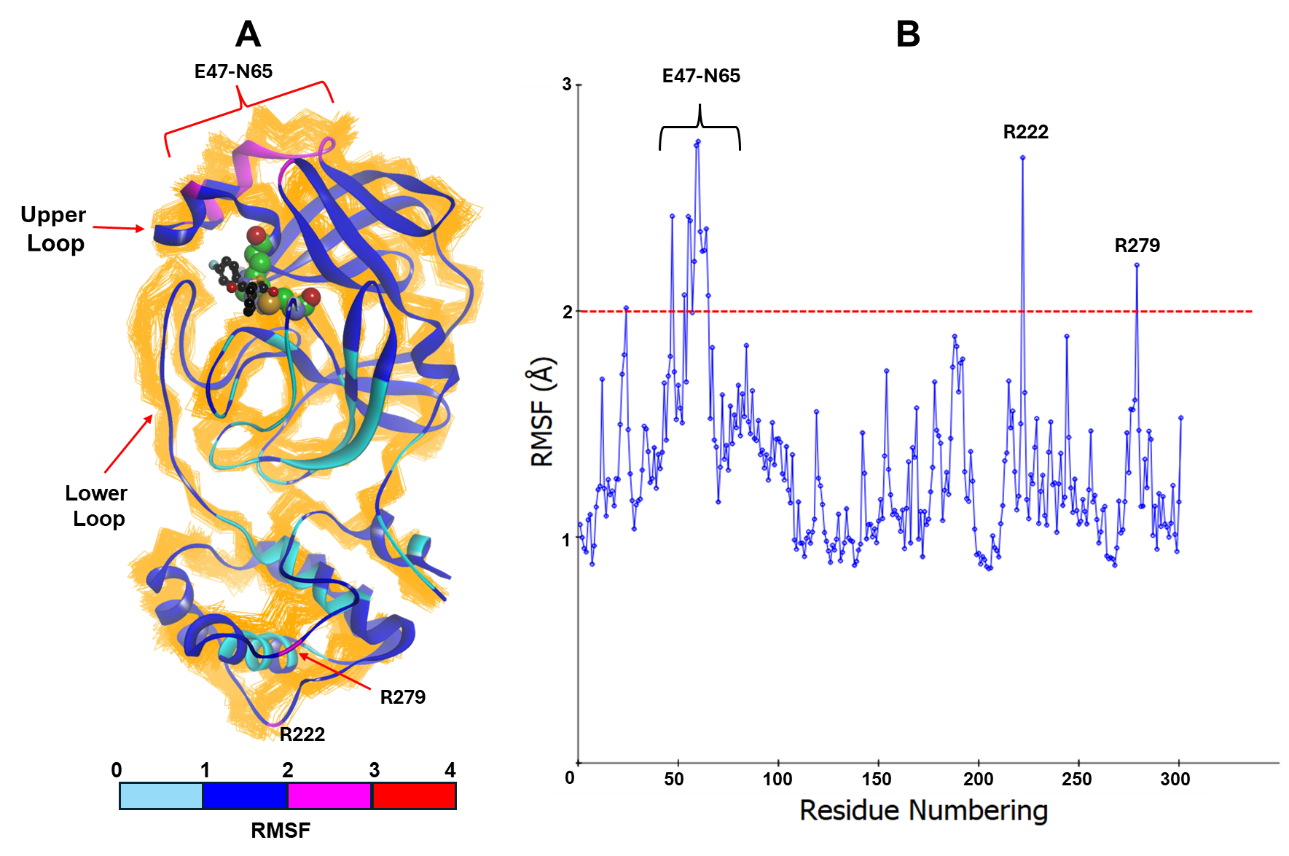
**

**Figure S12**. A) Steered Molecular Dynamics (SMD)results obtained for **THIA-10**. The resulting complexes were superimposed on the starting structure by using Cα. The starting complex is displayed as solid ribbons and colored according to the calculated RMSF values. The Cα of the resulting complexes are displayed as line ribbons and colored in orange. The ligand (black; ball&stick) and the catalytic residues (CPK and green) are colored by atom type (O: red; S: orange; N: blue; F: light blue). B) Mean Square Fluctuation (RMSF) values calculated considering all the resulting SMD structures. The residues with a RMSF value > 2 Å are labelled.


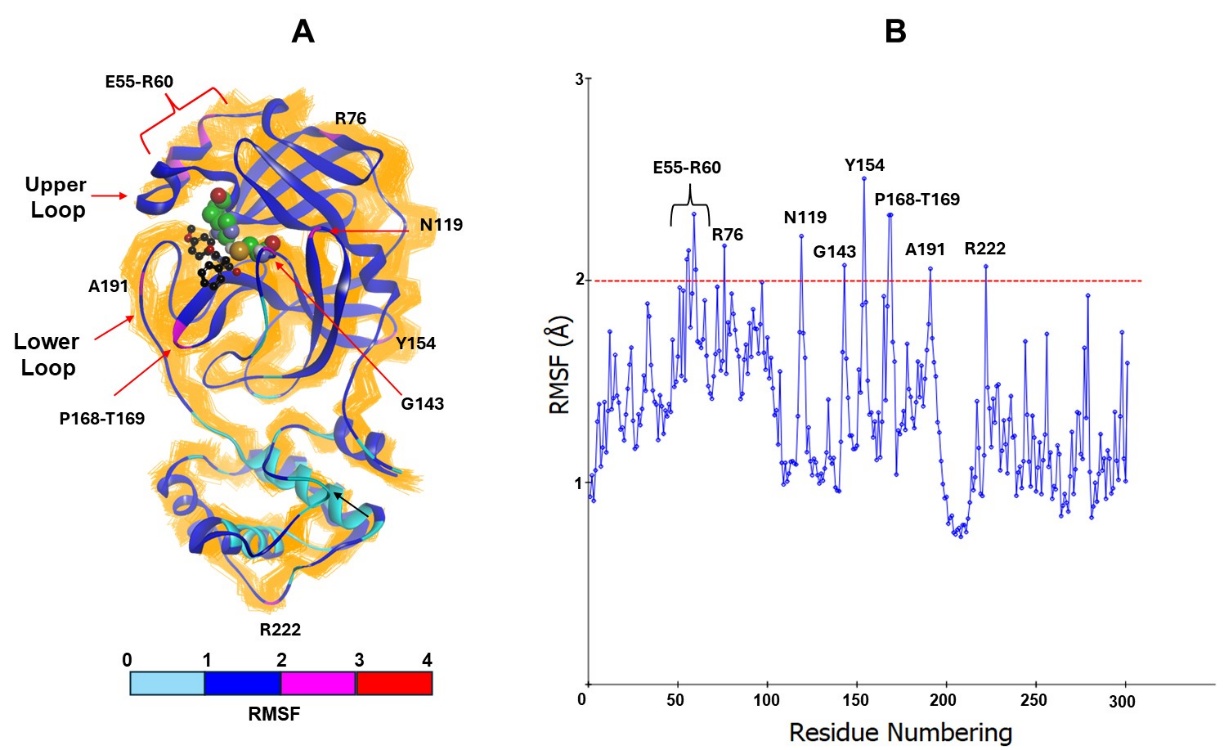


**Figure S13**. A) Steered Molecular Dynamics (SMD)results obtained for **THIA-2**. The resulting complexes were superimposed on the starting structure by using Cα. The starting complex is displayed as solid ribbons and colored according to the calculated RMSF values. The Cα of the resulting complexes are displayed as line ribbons and colored in orange. The ligand (black; ball&stick) and the catalytic residues (CPK and green) are colored by atom type (O: red; S: orange; N: blue). B) Mean Square Fluctuation (RMSF) values calculated considering all the resulting SMD structures. The residues with a RMSF value > 2 Å are labelled.


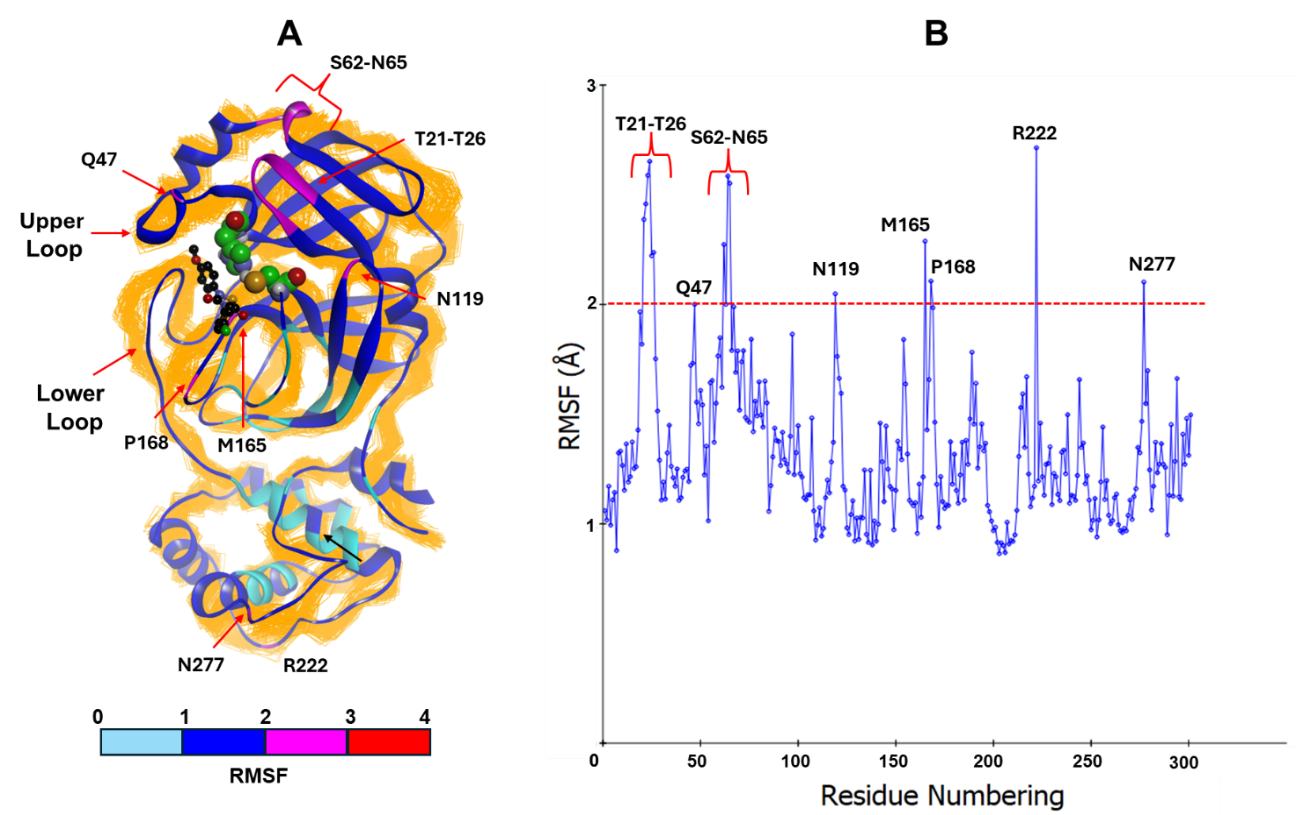


**Figure S14**. A) Steered Molecular Dynamics (SMD)results obtained for **THIA-7**. The resulting complexes were superimposed on the starting structure by using Cα. The starting complex is displayed as solid ribbons and colored according to the calculated RMSF values. The Cα of the resulting complexes are displayed as line ribbons and colored in orange. The ligand (black; ball&stick) and the catalytic residues (CPK and green) are colored by atom type (O: red; S: orange; N: blue; Cl: green). B) Mean Square Fluctuation (RMSF) values calculated considering all the resulting SMD structures. The residues with a RMSF value > 2 Å are labelled.


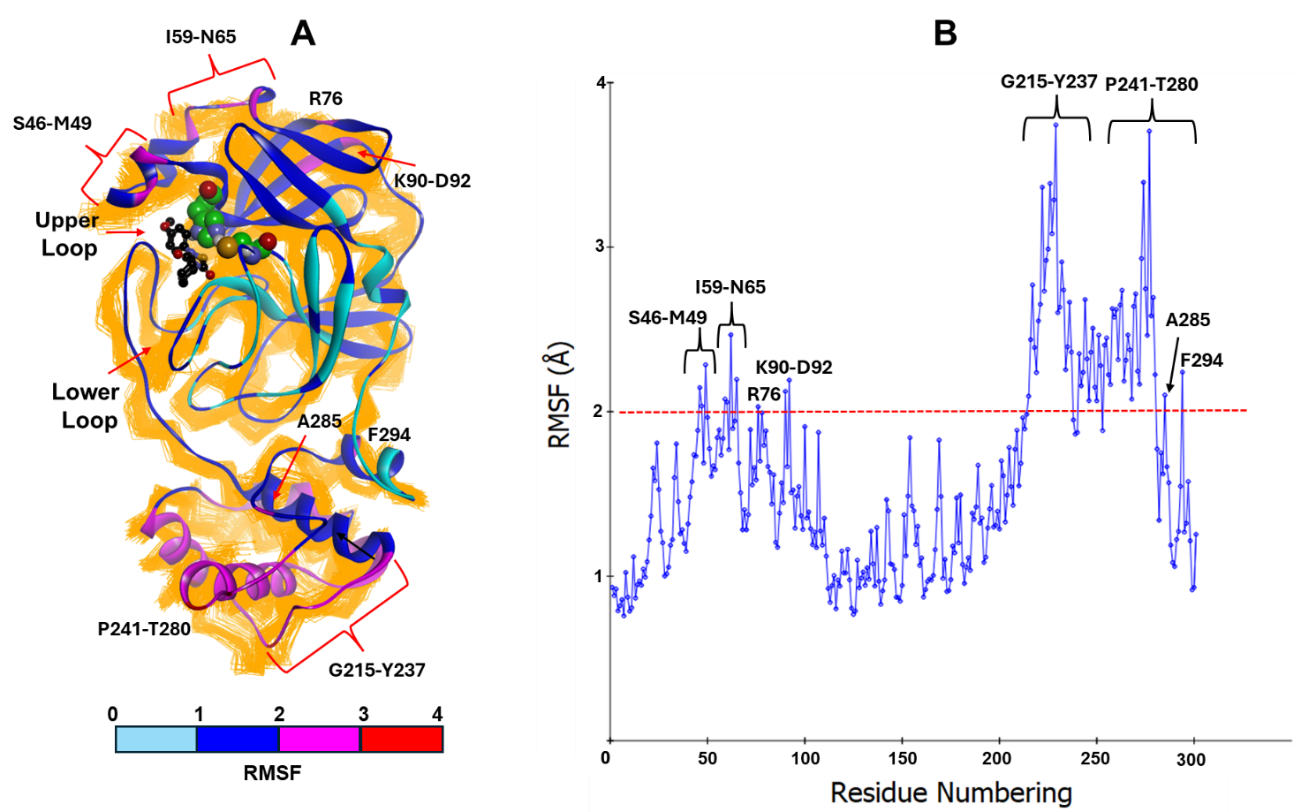


**Figure S15**. A) Steered Molecular Dynamics (SMD)results obtained for **THIA-8**. The resulting complexes were superimposed on the starting structure by using Cα. The starting complex is displayed as solid ribbons and colored according to the calculated RMSF values. The Cα of the resulting complexes are displayed as line ribbons and colored in orange. The ligand (black; ball&stick) and the catalytic residues (CPK and green) are colored by atom type (O: red; S: orange; N: blue). B) Mean Square Fluctuation (RMSF) values calculated considering all the resulting SMD structures. The residues with a RMSF value > 2 Å are labelled.


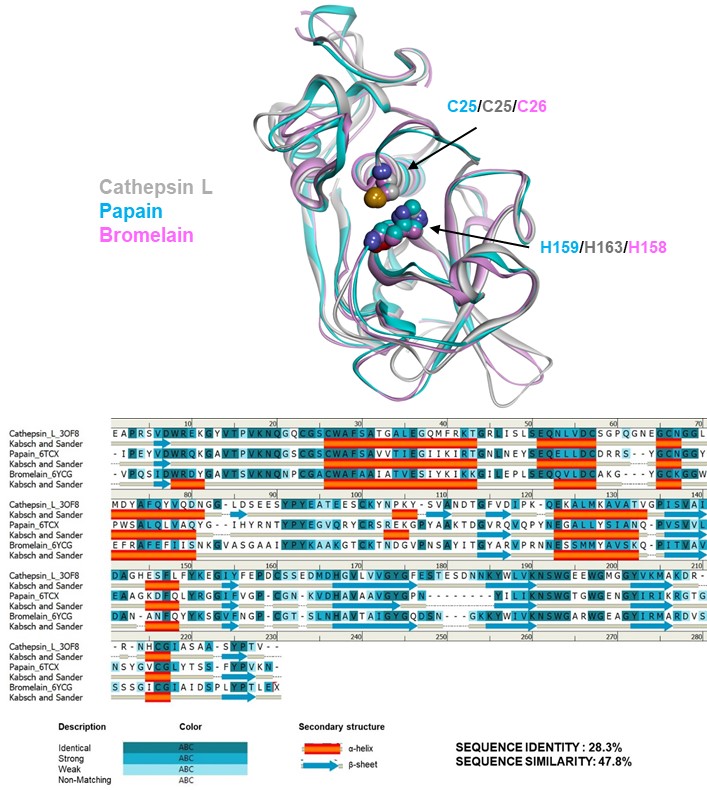


**Figure S16.** Up: superimposition among x-ray structure of Papain (cyan; PDB ID: 6TCX), x-ray structure of Cathepsin L (grey; PDB ID: 3OF8), and x-ray structure of Bromelain (pink; PDB ID: 6YCG). The proteins are displayed in solid ribbon. The catalytic residues Cys and His are shown in CPK and labelled. Bottom: secondary structure sequence alignment between Papain, Cathepsin L, and Bromelain. The sequence and the secondary structure assignments (Kabsch and Sander) are reported.


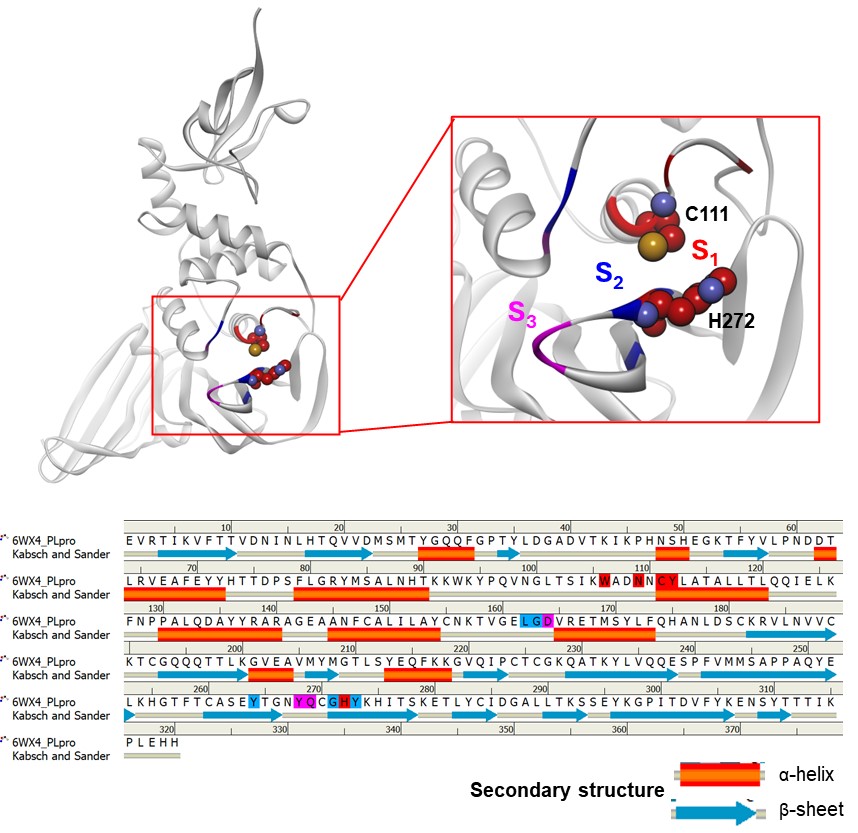


**Figure S17.** A: X-ray structure of SARS-CoV-2 PLpro (PDB ID: 6WX4). PLpro is displayed in solid ribbon and colored in grey. The residues of S_1_, S_2,_ and S_3_ subsites are coloured in red, blue, and magenta, respectively. The catalytic residues Cys and His are displayed in CPK and labelled. The sequence and the secondary structure assignments (Kabsch and Sander) are reported.


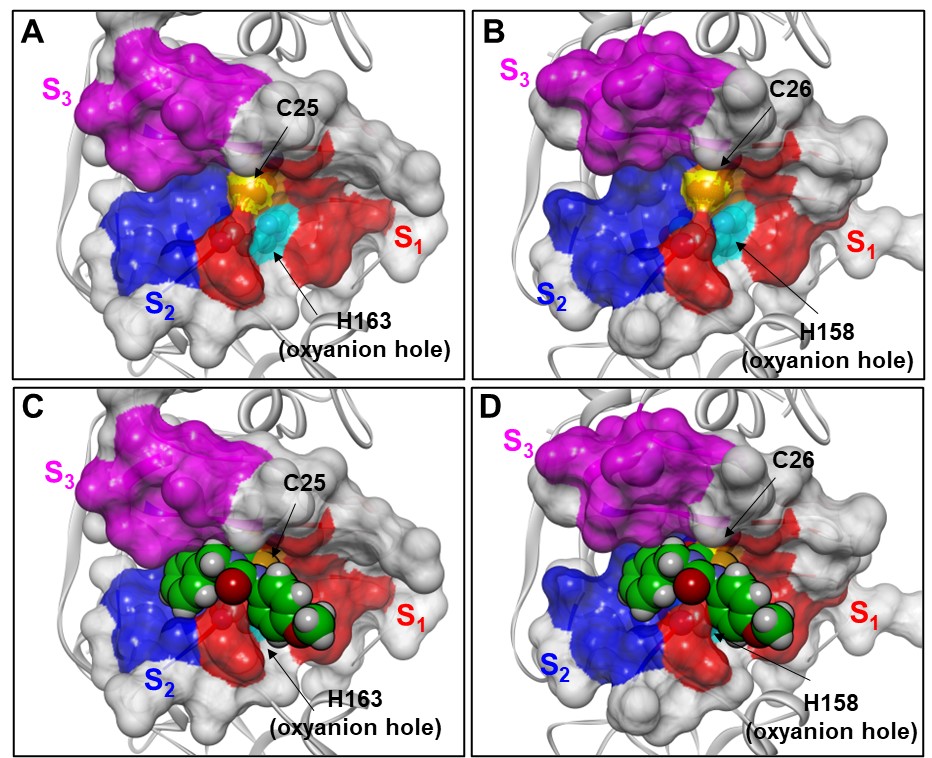


**Figure S18.** A: x-ray structure of Cathepsin L (PDB ID: 3OF8). B: X-ray structure of Bromelain (PDB ID: 6YCG). C: fitting of THIA-2 in the active site of Cathepsin L (PDB ID: 3OF8). D: fitting of THIA-2 in the active site of Bromelain (PDB ID: 6YCG). The proteins are displayed as grey ribbons. The catalytic residues Cys and His are shown in CPK, labelled and coloured in orange and cyan, respectively. The Connolly surfaces of the proteases are displayed in solid. The residues of S_1_, S_2,_ and S_3_ subsites are coloured in red, blue, and magenta, respectively. The solvent accessible surface (SASA) of the sulphur atom of the catalytic cysteine is displayed and coloured in yellow. THIA-2 is shown in CPK and coloured by atom type (C = green, O = red, N = blue, S = yellow).


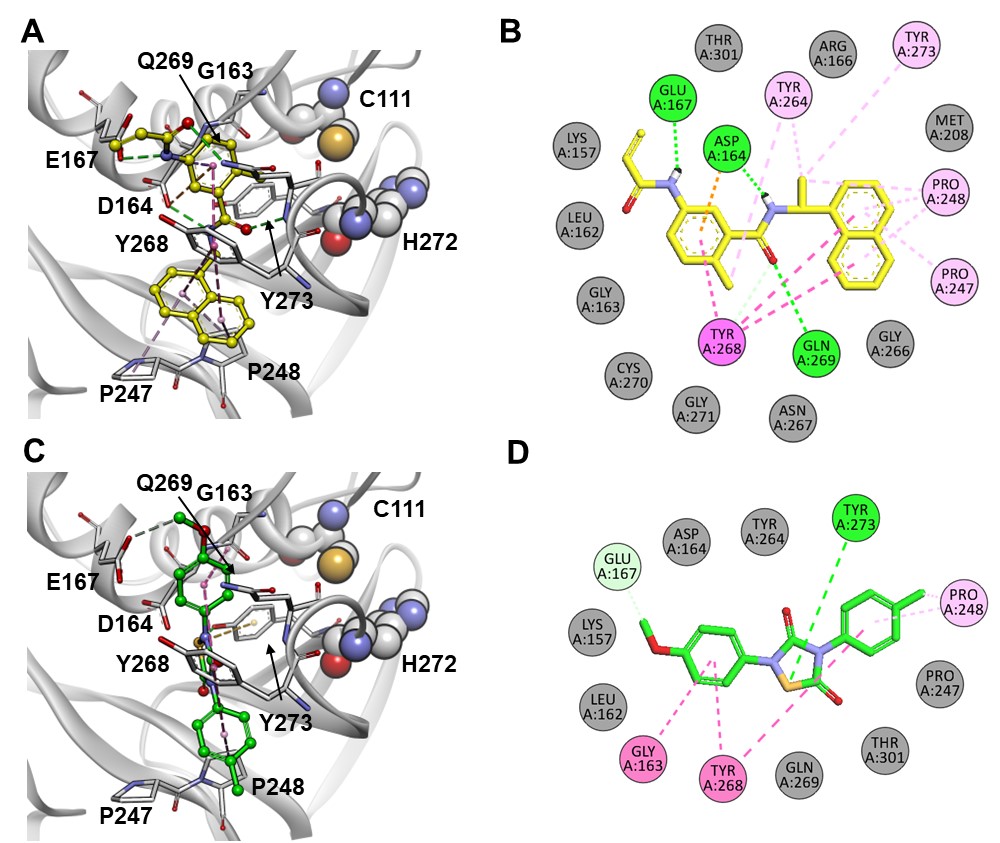


**Figure S19.** A: x-ray structure of SARS-CoV-2 PLpro in complex with a GRL0617 derivative (yellow; PDB ID: 7JIW). C: fitting of THIA-5 (green) in the allosteric site of PLpro (PDB ID:7JIW). The two catalytic residues (CPK) and the residues involved in the interactions (stick) are displayed and labelled. Ligands are displayed in ball&stick and coloured by atom type (O = red; N = blue; S = yellow). B and D: 2D representation of ligand-protein interactions (hydrogen bond: green; π-π interaction: magenta; alkyl interaction: pink; CH-O bond: light green).

**
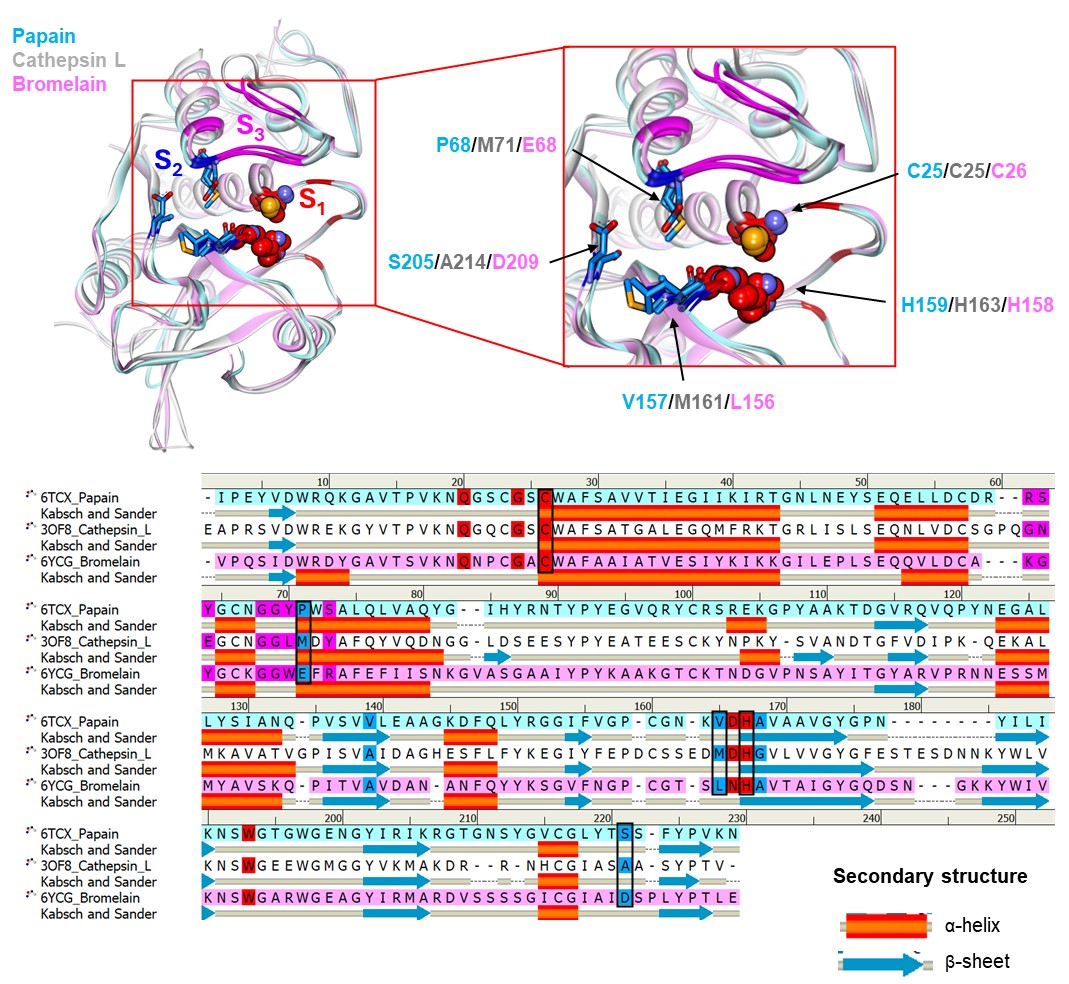
**

**Figure S20.** Up: superimposition among x-ray structure of Papain (cyan; PDB ID: 6TCX), x-ray structure of Cathepsin L (grey; PDB ID: 3OF8), and x-ray structure of Bromelain (pink; PDB ID: 6YCG). The proteins are displayed in solid ribbon. The residues of S_1_, S_2,_ and S_3_ subsites are coloured in red, blue, and magenta, respectively. The catalytic residues Cys and His are displayed in CPK and labelled. The different residues of S_2_ are displayed in stick and labelled. Bottom: secondary structure sequence alignment between Papain, Cathepsin L, and Bromelain. The sequence and the secondary structure assignments (Kabsch and Sander) are reported. A black square evidences the residues shown in the 3D image.


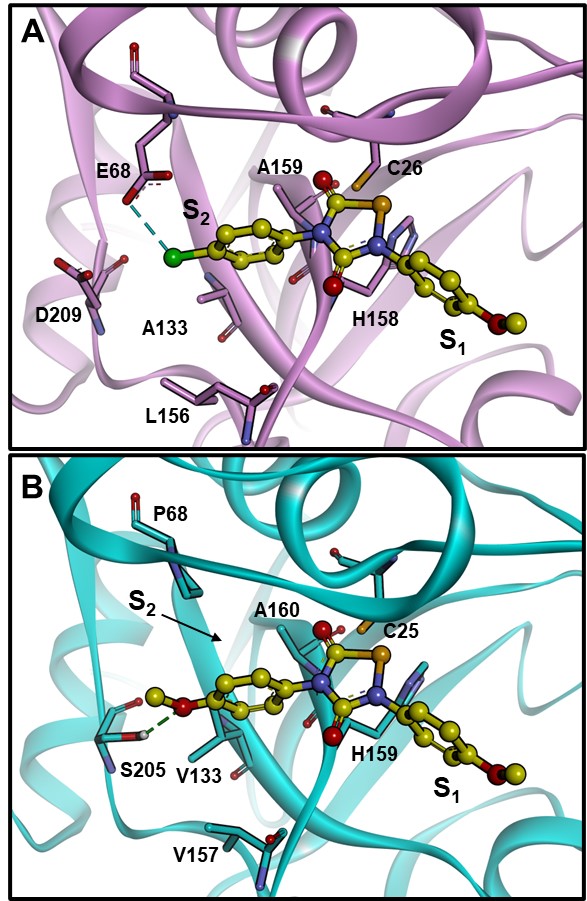


**Figure S21.** A: fitting of THIA-7 (yellow) in the active site of Bromelain (PDB ID:6YCG; pink). The hydrogen bond is displayed as a green dashed line. B: fitting of THIA-6 (yellow) in the active site of Papain (cyan). The halogen bond is displayed as a black dashed line. Papain and Bromelain are displayed as ribbons. Ligands are displayed in ball&stick and coloured by atom type (C= yellow; O=red; N=blue; S=orange; Cl=green). The catalytic residues and the residues of the S_2_ subsite are displayed in stick and labelled.
